# Design, Synthesis and Anti-Lung Cancer Evaluation of 1, 2, 3-Triazole Tethered Dihydroartemisinin-Isatin Hybrids

**DOI:** 10.3389/fphar.2021.801580

**Published:** 2021-12-16

**Authors:** Haodong Hou, Bin Qu, Chen Su, Guihua Hou, Feng Gao

**Affiliations:** Key Laboratory for Experimental Teratology of the Ministry of Education and Center for Experimental Nuclear Medicine, School of Basic Medical Sciences, Cheeloo College of Medicine, Shandong University, Jinan, China

**Keywords:** artemisinin, dihydroartemisinin, isatin, 1,2,3-triazole, hybrid molecules, multidrug resistance, structure-activity relationship

## Abstract

A series of 1,2,3-triazole tethered dihydroartemisinin-isatin hybrids 8a-c and 9a-k were designed and synthesized. Their antiproliferative activity against A549, doxorubicin-resistant A549 (A549/DOX) as well as cisplatin-resistant A549 (A549/DDP) lung cancer cell lines was also investigated in this study. All hybrids (half maximal inhibitory concentration/IC_50_: 7.54–73.8 *μ*M) were more potent than the parent drug dihydroartemisinin (IC_50_: 69.4–88.0 *μ*M) and also non-cytotoxic towards mouse embryonic fibroblast cells NIH/3T3 (IC_50_: >100 *μ*M). The structure-activity relationships illustrated that the substituents on C-3 and C-5 position of isatin moiety influenced the activity significantly. Imine at C-3 position decreased the activity, whereas fluoro at C-5 position enhanced the activity. In particular, hybrids 8a,c (IC_50_: 7.54–12.1 *μ*M) and 9i (IC_50_: 9.10–15.9 *μ*M) were comparable to cisplatin (IC_50_: 7.54–15.9 *μ*M *vs* 9.38–19.7 *μ*M) against A549 and A549/DOX, but 4.6–7.6 folds more potent than that of cisplatin (IC_50_: 8.77–14.3 *μ*M *vs* 66.9 *μ*M) against A549/DDP cells. Moreover, hybrids 8a,c exhibited excellent stability (liver microsomes: 68–83%) in mouse/human microsomes and good pharmacokinetic properties, demonstrating their potential as a novel anti-lung cancer chemotherapeutic candidates.

## Introduction

Lung cancer represents one of the most malignant tumors with the high morbidity and mortality, and non-small cell lung cancer (NSCLC, accounts for 80–85% of lung cancer cases) is the most aggressive type of lung cancer (Willis et al., 2019; Sławiński et al., 2020). Lung cancer is responsible for around 20% of all cancer deaths with an estimated 1.8 million new cases and 1.6 million deaths annually (Oak et al., 2012; Hirsch et al., 2017). The pace of the annual decline in lung cancer mortality doubled from 2.4% (during 2009 through 2013) to 5.0% (during 2014 through 2018) due to the advances in diagnostics and therapy, and this trend coincides with the steady declined incidence (2.2–2.3%) (Siegel et al., 2021; Wen et al., 2021). However, even lung cancer is diagnosed in the early stages, around one in four patients develop relapse and most of them die from recurrent disease (the overall 5-years survival rates are only around 15%) (Gray et al., 2019; Bade and Dela Cruz, 2020; Coakley and Popat, 2020; Schegoleva et al., 2021). Multidrug resistance, caused by various simulations such as off-target effect in G2/M arrest (Nascimento et al., 2017), insufficient production of apoptotic factors (Chen et al., 2014), and enhanced DNA repair (Nascimento et al., 2017; Kang et al., 2019), is also considered as a major challenge for cancer treatment (Yan et al., 2013; He et al., 2015; He et al., 2016). The absence of effective anti-lung cancer drugs, especially those against drug-resistant lung cancer, make the mortality of lung cancer still high. Therefore, it is urgent to develop novel drug candidates with high activity and efficacy against lung cancer, especially drug-resistant lung cancer.

**FIGURE 1:**
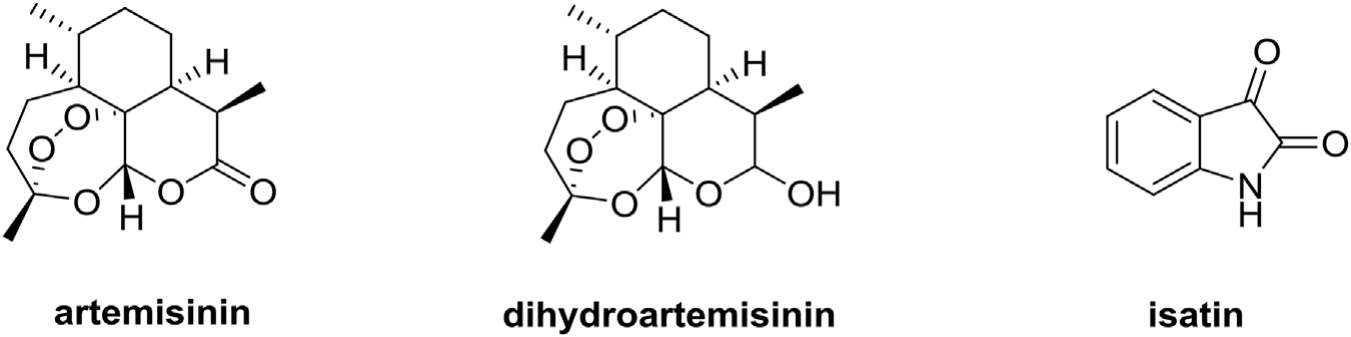
Chemical structures of artemisinin, dihydroartemisinin and isatin.

Artemisinin derivatives such as dihydroartemisinin (DHA, Figure 1) and artesunate, which own a unique sesquiterpene endoperoxide lactone moiety, could form highly reactive free radicals including reactive oxygen species (ROS) in the presence of ferrous ion (Fe^II^) (Yu et al., 2019; Gao et al., 2020). Fe^II^ accumulated in cancer cells is as much as 1,000 times that in normal cells, and artemisinin derivatives exhibit potential anticancer efficacy without significant cytotoxicity to normal cells, making these compounds far different from conventional chemotherapy (Dai et al., 2017; Kiani et al., 2020). The mechanistic studies elucidated that artemisinin derivatives could exert the anticancer activity *via* multiple mechanisms including inhibition of angiogenesis, apoptosis, cell cycle arrest, disruption of cell migration, and modulation of nuclear receptor responsiveness (Li et al., 2021; Zhu et al., 2021). Moreover, artemisinin derivatives could remarkably influence the growth of lung tumor *in vivo* through inhibiting Wnt/β-catenin pathway, revealing the potential application of artemisinin derivatives as a novel class of therapeutic drugs for lung cancer (Tong et al., 2016; Zhang et al., 2021).

Isatin derivatives have the potential to act on a variety of drug targets like histone deacetylase, *β*-carbonic anhydrase, tyrosine kinase and tubulin, and the isatin-based nintedanib has already been approved for the lung cancer therapy (Sharma et al., 2014; Ding et al., 2020; Hou et al., 2020; Nath et al., 2021; Varpe et al., 2021). Therefore, isatin derivatives are also considered as useful templates for the development of novel anti-lung cancer agents.

Molecular hybridization represents one of the common strategies to discover new drugs since hybrid molecules usually own dual/multiple modes of action that can overcome drug resistance, enhance the efficacy, reduce adverse effects, and improve pharmacokinetic and pharmacodynamic properties (Singh et al., 2013; Nepali et al., 2014; Saadeh and Mubarak, 2017; Feng et al., 2020). Accordingly, hybridization of dihydroartemisinin with isatin may open a door to develop potential drug candidates against lung cancers including drug-resistant forms.

**FIGURE 2:**
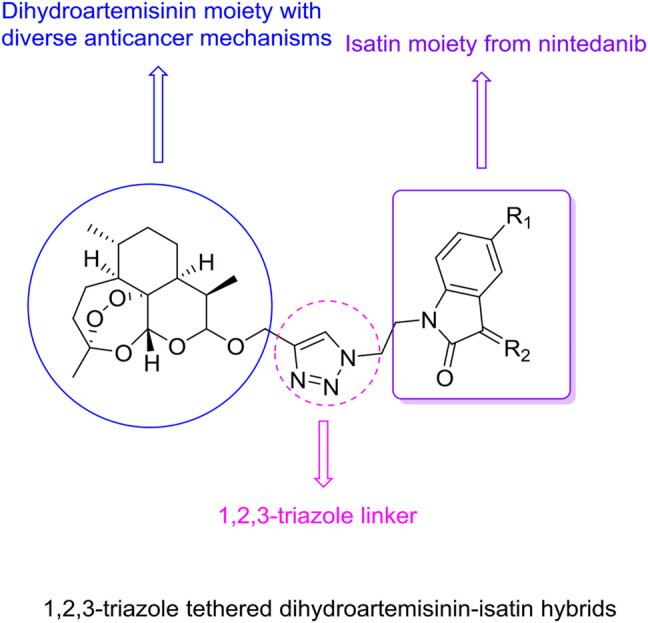
Chemical structures of 1,2,3-triazole tethered dihydroartemisinin-isatin hybrids.

It is reported triazoles hold potential cytotoxic towards cancer cells, which attracted us towards the selection of 1,2,3-triazole as a linker between the two functionalities (Sharma et al., 2015; Singh et al., 2016; Singh et al., 2017). Herein, we report the design, synthesis of various novel 1,2,3-triazole tethered dihydroartemisinin-isatin hybrids (Figure 2), and evaluation of their *in vitro* antiproliferative activity against A549, doxorubicin-resistant A549 (A549/DOX), cisplatin-resistant A549 (A549/DDP) lung cancer cell lines, cytotoxicity towards mouse embryonic fibroblast cells NIH/3T3, liver stability and pharmacokinetic properties in this study. Our major goal is to optimize the anti-lung cancer potency of these hybrids, and preliminary studies on structure-activity relationships (SARs) are also taken to facilitate the further development of these hybrids.

## Results and Discussion

### Synthesis

**Scheme 1:**
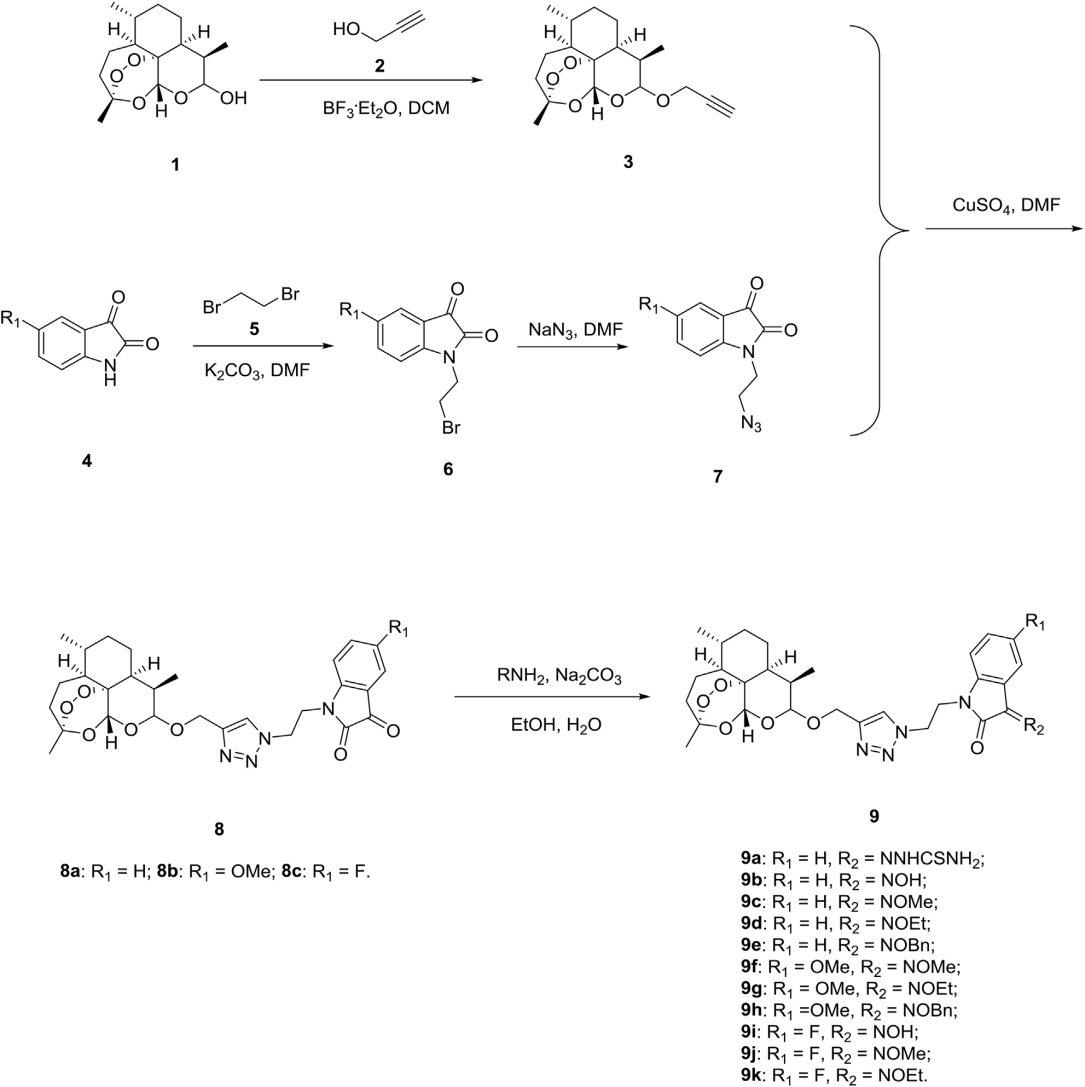
Synthesis of 1,2,3-triazole tethered dihydroartemisinin-isatin hybrids 8a-c and 9a-k

Firstly, we designed and synthesized the desired 1,2,3-triazole tethered dihydroartemisinin-isatin hybrids 8a-c and 9a-k following the synthetic routes shown in Scheme 1. Dihydroartemisinin 1 reacted with propargyl alcohol 2 in presence of boron trifluoride diethyl etherate (BF_3_^**.**^ OEt_2_) and yielded alkynyl-containing dihydroartemisinin intermediate 3. Alkylation between isatins 4 and 1,2-dibromoethane 5) with potassium carbonate (K_2_CO_3_) as base generated intermediates 6, which were then reacted with sodium azide to give azido precursors 7. The desired 1,2,3-triazole tethered dihydroartemisinin-isatin hybrids 8a-c were obtained through Cu-promoted azide-alkyne cycloaddition reaction between intermediate 3 and azido precursors 7. Finally, 1,2,3-triazole tethered dihydroartemisinin-isatin hybrids 8a-c reacted with amine hydrochlorides using sodium carbonate (Na_2_CO_3_) as base and provided desired hybrids 9a-k.

All of the desired 1,2,3-triazole tethered dihydroartemisinin-isatin hybrids 8a-c and 9a-k were characterized by MS, ^1^H NMR and ^13^C NMR, and the corresponding analytical spectra were in the supplementary information section. The chemical structures and yields of desired hybrids were listed in Table 1.

**TABLE 1:** Chemical structures and yields of 1,2,3-triazole tethered dihydroartemisinin-isatin hybrids 8a-c and 9a-k.
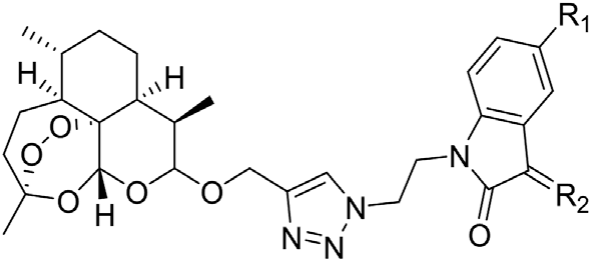

Compd	R_1_	R_2_	Yield (%)
**8a**	H	O	37
**8b**	OMe	O	28
**8c**	F	O	33
**9a**	H	NNHCSNH_2_	49
**9b**	H	NOH	83
**9c**	H	NOMe	62
**9d**	H	NOEt	57
**9e**	H	NOBn	48
**9f**	OMe	NOMe	59
**9g**	OMe	NOEt	42
**9h**	OMe	NOBn	41
**9i**	F	NOMe	67
**9j**	F	NOMe	52
**9k**	F	NOEt	43

### The *In Vitro* Antiproliferative Activity and Cytotoxicity

The antiproliferative activity of 1,2,3-triazole tethered dihydroartemisinin-isatin hybrids 8a-c and 9a-k against A549, multidrug-resistant A549/DOX and A549/DDP lung cancer cell lines as well as cytotoxicity towards mouse embryonic fibroblast cells NIH/3T3 were assessed by 3-(4,5-dimethylthiazol-2-yl)-2,5-diphenyltetrazolium bromide (MTT) assay, and half maximal inhibitory concentration (IC_50_) values were listed in Table 2.

**TABLE 2:** The antiproliferative activities, cytotoxicity, selectivity index and resistance index values of 1,2,3-triazole tethered dihydroartemisinin-isatin hybrids **8a-c** and **9a-k**.

Compd	IC_50_ (*μ*M)				SI^a^	RI	
	A549	A549/DOX^b^	A549/DDP^c^	NIH/3T3		RI1^d^	RI2^e^
**8a**	8.32	12.1	10.7	>100	>12.0	1.45	1.29
**8b**	21.6	32.0	19.5	>100	>4.6	1.48	0.90
**8c**	7.54	9.89	8.77	>100	>13.2	1.31	1.16
**9a**	16.3	38.4	30.9	>100	>6.1	2.35	1.90
**9b**	12.0	26.1	30.3	>100	>8.3	2.18	2.52
**9c**	22.7	19.5	27.4	>100	>4.4	0.86	1.20
**9d**	25.6	20.8	33.7	>100	>3.9	0.81	1.32
**9e**	16.3	31.4	29.9	>100	>6.1	1.92	1.83
**9f**	65.4	79.5	51.8	>100	>1.5	1.22	0.79
**9g**	73.8	45.0	61.7	>100	>1.3	0.61	0.84
**9h**	44.7	77.7	59.4	>100	>2.2	1.74	1.33
**9i**	9.10	15.9	14.3	>100	>11.0	1.75	1.57
**9j**	9.61	13.0	38.2	>100	>10.4	1.35	3.98
**9k**	15.8	31.1	28.7	>100	>6.3	1.97	1.82
Artemisinin	>100	>100	>100	>100	-	-	-
DHA^f^	69.4	88.0	75.9	>100	>1.4	1.27	1.09
cisplatin	9.38	19.7	66.9	>100	>10.6	2.10	7.13

b Doxorubicin-resistant A549 cells.

c Cisplatin-resistant A549 cells.

a Selectivity index: IC_50(NIH/3T3)_/IC_50(A549)_.

d Resistance index: IC_50(A549/DOX)_/IC_50(A549)_.

e Resistance index: IC_50(A549/DDP)_/IC_50(A549)_.

f Dihydroartemisinin.

From Table 2, it can be concluded that all of the desired 1,2,3-triazole tethered dihydroartemisinin-isatin hybrids 8a-c and 9a-k were active against A549, multidrug-resistant A549/DOX and A549/DDP lung cancer cell lines with IC_50_ values of 7.54–73.8 *µ*M. All hybrids were superior to the reference drugs artemisinin (IC_50_: >100 *µ*M) and DHA (IC_50_: 69.4–88.0 *µ*M) against both drug-sensitive and multidrug-resistant A549 lung cancer cell lines, and some of them also possessed higher activity than cisplatin (IC_50_: 9.38–66.9 *µ*M). The SAR illustrated that introduction of hydroxime, alkyloxime, benzyloxime and thiosemicarbazide into C-3 position of isatin moiety reduced the activity in comparison with the carbonyl analogs. Substituents on the C-5 position of isatin motif had significant influence on the activity, and electron-withdrawing fluoro was beneficial for the activity, while electron-donating methoxy group led to great loss of activity.

All the desired hybrids (IC_50_: >100 *μ*M) were non-cytotoxic towards mouse embryonic fibroblast cells NIH/3T3, and the selectivity index (SI: IC_50(NIH/3T3)_/IC_50(A549)_) values were >1.3, implying that these hybrids possessed acceptable specifity. The desired hybrids showed the same level activity against both drug-sensitive and multidrug-resistant A549 lung cancer cell lines, and the drug resistance index (RI: IC_50(MDR A549)_/IC_50(A549)_) values were 0.61–3.98, revealing that these hybrids had low level cross resistance with doxorubicin and cisplatin.

Among them, the representative hybrids 8a,c (IC_50_: 7.54–12.1 *μ*M) and 9i (IC_50_: 9.10–15.9 *μ*M) were highly potent against the three tested lung cancer cell lines, and the activity was comparable to that of cisplatin (IC_50_: 7.54–15.9 *μ*M *vs* 9.38–19.7 *μ*M) against A549 and A549/DOX, but 4.6–7.6 folds higher than that of cisplatin (IC_50_: 8.77–14.3 *μ*M *vs* 66.9 *μ*M) against A549/DDP cells.

The metabolic stability of selected hybrids 8a,c and 9i was assessed in mouse and human microsomes, and the results were listed in Table 3. It can be concluded that hybrids 8a,c (liver microsomes: 68–83%) with carbonyl group at C-3 position of isatin moiety exhibited a superior microsomal stability than the hydroxime analog 9i (liver microsomes: 46 and 60%).

**TABLE 3:** Stability of selected hybrids **8a,c** and **9i** in mouse and human microsomes.

Compd	Liver microsomes [%]	
	Mouse	Human
**8a**	77	68
**8c**	71	83
**9i**	46	60

The pharmacokinetic behavior of hybrids 8a,c was determined in CD-1 mice model by single intravenous administration with dose of 30 mg/kg. As summarized in Table 4, the pharmacokinetic properties of hybrids **8a,c** as follows: the maximum plasma concentrations (*C*_max_) of 6.4 and 12.5 *μ*M, area under curve (AUC) of 883 and 654 ng h/ml, clearance rates (Cl) of 2.31 and 3.16 L/h/kg, half-lives (t_1/2_) of 3.7 and 4.2 h, peak time of 12 min, and bioavailability of 35.6 and 27.5%.

**TABLE 4:** Pharmacokinetic properties of hybrids **8a,c** in mice.

Parameter	Compd.	
	8a	8c
*C*_max_ (*μ*M)	6.4	12.5
AUC (ng^**.**^h/ml)	883	654
t_1/2_ (h)	3.7	4.2
t_max_ (min)	12	12
Cl (L/h/kg)	2.31	3.16
*F* (%)	35.6	27.5

## Conclusion

A series of 1,2,3-triazole tethered dihydroartemisinin-isatin hybrids 8a-c and 9a-k were designed, synthesized and assessed for their antiproliferative activity against A549, A549/DOX, and A549/DDP lung cancer cell lines in this study. All of these hybrids (IC_50_: 7.54–73.8 *μ*M) were more potent than the parent drug dihydroartemisinin (IC_50_: 69.4–88.0 *μ*M) against the tested cancer cell lines. In addition, all hybrids (IC_50_: >100 *μ*M) displayed non-cytotoxic towards NIH/3T3 cells. Among them, hybrids 8a,c (IC_50_: 7.54–12.1 *μ*M) and 9i (IC_50_: 9.10–15.9 *μ*M) were not inferior to cisplatin (IC_50_: 9.38–66.9 *μ*M) against the three cancer cell lines. Moreover, hybrids 8a,c possessed excellent stability and good pharmacokinetic properties, demonstrating their potential as novel anti-lung cancer chemotherapeutic candidates. Accordingly, hybrids 8a,c merits further preclinical evaluations.

## Experimental Section

### Materials

^1^H NMR and ^13^C NMR spectra were determined on a Varian Mercury-400 spectrometer in CDCl^3^fn3 using tetramethylsilane (TMS) as an internal standard. Electrospray ionization (ESI) mass spectra were obtained on a MDSSCIEXQ-Tap mass spectrometer. Unless otherwise noted, the reagents were obtained from commercial supplier and were used without further purification. A549, A549/DOX, and A549/DDP lung cancer cell lines were purchased from the American Type Culture Collection (ATCC) and preserved by Center for Experimental Nuclear Medicine of Shandong University.

### Synthesis

To a mixture of dihydroartemisinin 1 (100 mmol) and propargyl alcohol 2 (120 mmol) in DCM (500 ml) was added boron trifluoride diethyl etherate (BF_3_^**.**^ OEt_2_, 20 ml) at 0°C, and the mixture was stirred at room temperatire overnight. Sat. Na_2_CO_3_ (500 ml) was added to the mixture, and then the organic layer was separated. The organic layer was washed with H_2_O (500 ml) and brine (500 ml) in sequence, dried over anhydrous Na_2_SO_4_, filtered, and concentrated under reduced pressure to give crude alkynyl-containing dihydroartemisinin intermediate 3.

To a solution of isatins 4 (100 mmol) in DMF (100 ml), potassium carbonate (K_2_CO_3_, 200 mmol) was added. The mixture was stirred at room temperature for 1 h, and then 1,2-dibromoethane (5, 150 mmol) was added. The mixture was stirred overnight at room temperature, and then filtered. The mixture was concentrated under reduced pressure and the residue was purified by silica gel chromatography eluted with PE to PE:EA = 2:1 to provide intermediates 6.

A mixture of intermediates 6 (10 mmol) and NaN_3_ (15 mmol) in DMF (30 mmol) was stirred at 50°C for 12 h, and then cooled to room temperature. H_2_O (100 ml) was added to the mixture, and the mixture was extracted with DCM (100 ml × 3). The combined organic layers were washed with H_2_O (500 ml) and brine (500 ml) in sequence, dried over anhydrous Na_2_SO_4_, filtered, and concentrated under reduced pressure to give crude azido precursors 7.

The mixture of intermediates 6 (3 mmol), precursors 7 (3 mmol) and CuSO_4_ (1 mmol) in DMF (10 mmol) was stirred at 60°C for 8 h under N_2_ atmosphere, and then cooled to room temperature. After filtration, the filtrate was concentrated under reduced pressure. The residue was purified by silica gel chromatography eluted with PE to PE:EA = 1:2 to generate 1,2,3-triazole tethered dihydroartemisinin-isatin hybrids 8a-c.

To a solution of hybrids 8a-c (1 mmol) and amine hydrochlorides (1.5 mmol) in a mixture of EtOH (10 ml) and H_2_O (10 ml), Na_2_CO_3_ (2 mmol) was added. The mixture was stirred at 60°C for 12 h, and then cooled to room temperature. The mixture was extracted with DCM (20 ml × 3). The combined organic layers were washed with H_2_O (30 ml) and brine (30 ml) in sequence, dried over anhydrous Na_2_SO_4_, filtered, and concentrated under reduced pressure. The residue was purified by silica gel chromatography eluted with PE to PE:EA = 1:2 to give 1,2,3-triazole tethered dihydroartemisinin-isatin hybrids 9a-k.

### Characterization

1-(2-(4-((((3*R*,5a*S*,6*R*,8a*S*,9*R*,12*R*,12a*R*)-3,6,9-trimethyldecahydro-12*H*-3,12-epoxy (Willis et al., 2019; Sławiński et al., 2020)dioxepino [4,3-i]isochromen-10-yl)oxy)methyl)-1*H*-1,2,3-triazol-1-yl)ethyl)indoline-2,3-dione (**8a**).

Red solid, yield: 37%. ^1^H NMR (400 MHz, CDCl_3_) δ 0.75–0.92 (m, 7H), 1.11–1.14 (m, 1H), 1.31–1.35 (m, 1H), 1.48–1.55 (m, 5H), 1.60–1.63 (m, 1H), 1.68–1.71 (m, 2H), 1.76–1.80 (m, 2H), 1.99–2.06 (m, 1H), 2.33–2.36 (m, 1H), 3.50 (d, *J* = 4.0 Hz, 1H), 4.18 (t, *J* = 4.0 Hz, 2H), 4.46 (d, *J* = 8.0 Hz, 1H), 4.69 (t, *J* = 4.0 Hz, 2H), 4.74 (dd, *J* = 8.0, 4.0 Hz, 1H), 5.18 (s, 1H), 6.52 (d, *J* = 4.0 Hz, 1H), 7.00 (d, *J* = 4.0 Hz, 1H), 7.42 (t, *J* = 4.0 Hz, 1H), 7.50 (d, *J* = 4.0 Hz, 1H). ^13^C NMR (100 MHz, CDCl_3_) δ 182.36, 158.57, 150.05, 145.72, 138.69, 125.61, 124.20, 123.54, 117.38, 109.63, 108.02, 99.51, 93.67, 84.12, 69.56, 61.51, 47.70, 42.47, 40.73, 40.59, 34.83, 34.67, 30.33, 30.23, 25.00, 21.00, 18.82, 12.31. HRMS-ESI: m/z Calcd for C_28_H_34_N_4_O_7_Na [M + Na]^+^: 561.2320; Found: 561.2314.

5-methoxy-1-(2-(4-((((3*R*,5a*S*,6*R*,8a*S*,9*R*,12*R*,12a*R*)-3,6,9-trimethyldecahydro-12*H*-3,12-epoxy (Willis et al., 2019; Sławiński et al., 2020)dioxepino [4,3-i]isochromen-10-yl)oxy)methyl)-1*H*-1,2,3-triazol-1-yl)ethyl)indoline-2,3-dione (8b).

Red solid, yield: 28%. ^1^H NMR (400 MHz, CDCl_3_) δ 0.75–0.92 (m, 7H), 1.13–1.17 (m, 1H), 1.30–1.35 (m, 1H), 1.48–1.56 (m, 5H), 1.60–1.63 (m, 1H), 1.68–1.70 (m, 2H), 1.76–1.81 (m, 2H), 1.98 (d, *J* = 8.0 Hz, 1H), 2.34–2.36 (m, 1H), 3.50 (d, *J* = 4.0 Hz, 1H), 3.70 (s, 3H), 4.15 (t, *J* = 4.0 Hz, 2H), 4.48 (d, *J* = 12.0 Hz, 1H), 4.62 (t, *J* = 4.0 Hz, 2H), 4.70 (dd, *J* = 2.0 Hz, 1H), 4.74 (d, *J* = 4.0 Hz, 1H), 5.18 (s, 1H), 6.44 (d, *J* = 4.0 Hz, 1H), 6.94 (dd, *J* = 4.0, 2.0 Hz, 1H), 7.02 (d, *J* = 4.0 Hz, 1H), 7.20 (s, 1H), 7.93 (s, 1H). ^13^C NMR (100 MHz, CDCl_3_) δ 182.72, 158.70, 156.70, 145.72, 143.88, 135.79, 125.52, 124.80, 123.53, 117.84, 110.68, 109.80, 108.02, 99.49, 93.70, 84.12, 69.58, 61.52, 55.93, 47.78, 42.47, 40.78, 40.61, 34.83, 34.68, 30.33, 30.25, 24.99, 20.99, 18.82, 12.27. HRMS-ESI: m/z Calcd for C_29_H_36_N_4_O_8_Na [M + Na]^+^: 591.2426; Found: 591.2410.

5-fluoro-1-(2-(4-((((3*R*,5a*S*,6*R*,8a*S*,9*R*,12*R*,12a*R*)-3,6,9-trimethyldecahydro-12*H*-3,12-epoxy (Willis et al., 2019; Sławiński et al., 2020)dioxepino [4,3-i]isochromen-10-yl)oxy)methyl)-1*H*-1,2,3-triazol-1-yl)ethyl)indoline-2,3-dione (8c).

Red solid, yield: 33%. ^1^H NMR (400 MHz, CDCl_3_) δ 0.76–0.93 (m, 7H), 1.13–1.14 (m, 1H), 1.33–1.35 (m, 1H), 1.48–1.56 (m, 5H), 1.60–1.63 (m, 1H), 1.68–1.71 (m, 3H), 1.76–1.82 (m, 2H), 1.98–2.06 (m, 1H), 2.34–2.37 (m, 1H), 3.50 (s, 1H), 4.20 (t, *J* = 4.0 Hz, 2H), 4.46 (d, *J* = 8.0 Hz, 1H), 4.63 (t, *J* = 4.0 Hz, 2H), 4.80 (dd, *J* = 8.0, 4.0 Hz, 1H), 5.18 (s, 1H), 6.50 (dd, *J* = 8.0, 4.0 Hz, 1H), 7.12 (td, *J* = 8.0, 2.0 Hz, 1H), 7.20 (dd, *J* = 4.0, 2.0 Hz, 1H), 7.44 (s, 1H). ^13^C NMR (100 MHz, CDCl_3_) δ 181.80, 160.19 (*J* = 205.00 Hz), 158.35, 146.13, 145.79, 127.10, 124.94, 123.63, 118.01, 117.97, 112.65, 112.49, 111.03, 110.98, 108.03, 99.47, 93.75, 84.08, 69.56, 61.53, 47.74, 42.42, 40.88, 40.57, 34.84, 34.65, 30.32, 30.24, 24.99, 20.97, 18.81, 12.22. HRMS-ESI: m/z Calcd for C_28_H_33_FN_4_O_7_Na [M + Na]^+^: 579.2226; Found: 579.2233.

2-(2-oxo-1-(2-(4-((((3*R*,5a*S*,6*R*,8a*S*,9*R*,12*R*,12a*R*)-3,6,9-trimethyldecahydro-12*H*-3,12-epoxy (Willis et al., 2019; Sławiński et al., 2020)dioxepino [4,3-i]isochromen-10-yl)oxy)methyl)-1*H*-1,2,3-triazol-1-yl)ethyl)indolin-3-ylidene)hydrazine-1-carbothioamide (9a).

Yellow solid, yield: 49%. ^1^H NMR (400 MHz, CDCl_3_) δ 0.75–0.92 (m, 7H), 1.12–1.16 (m, 1H), 1.31–1.36 (m, 1H), 1.48–1.56 (m, 5H), 1.60–1.62 (m, 1H), 1.69–1.72 (m, 2H), 1.72–1.81 (m, 2H), 2.05–2.06 (m, 1H), 2.33–2.36 (m, 1H), 3.50 (d, *J* = 2.0 Hz, 1H), 4.20 (td, *J* = 4.0, 2.0 Hz, 2H), 4.46 (d, *J* = 8.0 Hz, 1H), 4.61–4.65 (m, 2H), 4.67 (d, *J* = 2.0 Hz, 1H), 4.72 (d, *J* = 8.0, 4.0 Hz, 1H), 5.18 (s, 1H), 6.52 (d, *J* = 4.0 Hz, 1H), 6.75 (s, 1H), 7.00 (t, *J* = 4.0 Hz, 1H), 7.22 (t, *J* = 4.0 Hz, 1H), 7.38 (s, 1H), 7.46 (d, *J* = 4.0 Hz, 1H), 7.49 (s, 1H), 12.60 (s, 1H). ^13^C NMR (100 MHz, CDCl_3_) δ 179.92, 161.24, 145.64, 142.37, 131.76, 131.15, 123.69, 123.37, 121.01, 119.06, 108.84, 108.02, 99.32, 93.71, 84.13, 69.58, 61.40, 47.72, 42.45, 40.59, 40.36, 34.83, 34.70, 30.34, 30.25, 25.01, 21.01, 18.84, 12.31. HRMS-ESI: m/z Calcd for C_29_H_37_N_7_O_6_SNa [M + Na]^+^: 634.2419; Found: 634.2407.

3-(hydroxyimino)-1-(2-(4-((((3*R*,5a*S*,6*R*,8a*S*,9*R*,12*R*,12a*R*)-3,6,9-trimethyldecahydro-12*H*-3,12-epoxy (Willis et al., 2019; Sławiński et al., 2020)dioxepino [4,3-i]isochromen-10-yl)oxy)methyl)-1*H*-1,2,3-triazol-1-yl)ethyl)indolin-2-one (9b).

Yellow solid, yield: 83%. ^1^H NMR (400 MHz, CD_3_OD) δ 0.71–0.90 (m, 7H), 1.07–1.20 (m, 2H), 1.36–1.41 (m, 4H), 1.49–1.56 (m, 3H), 1.67–1.70 (m, 2H), 1.74–1.78 (m, 1H), 2.26–2.29 (m, 1H), 3.40 (d, *J* = 2.0 Hz, 1H), 4.16 (t, *J* = 4.0 Hz, 1H), 4.40 (d, *J* = 8.0 Hz, 1H), 4.57–4.68 (m, 4H), 5.15 (s, 1H), 6.66 (d, *J* = 4.0 Hz, 1H), 6.96 (t, *J* = 8.0 Hz, 1H), 7.22 (d, *J* = 8.0 Hz, 1H), 7.78 (s, 1H), 7.92 (d, *J* = 4.0 Hz, 1H). ^13^C NMR (100 MHz, CD_3_OD) δ 164.80, 144.81, 143.34, 142.37, 131.67, 127.21, 124.47, 123.04, 115.55, 108.17, 107.99, 98.98, 93.50, 83.80, 68.59, 60.31, 42.08, 40.59, 39.92, 34.53, 34.49, 30.20, 30.08, 24.81, 20.09, 17.92, 11.34. HRMS-ESI: m/z Calcd for C_30_H_39_FN_5_O_7_Na [M + Na]^+^: 576.2429; Found: 576.2440.

3-(methoxyimino)-1-(2-(4-((((3*R*,5a*S*,6*R*,8a*S*,9*R*,12*R*,12a*R*)-3,6,9-trimethyldecahydro-12*H*-3,12-epoxy (Willis et al., 2019; Sławiński et al., 2020)dioxepino [4,3-i]isochromen-10-yl)oxy)methyl)-1*H*-1,2,3-triazol-1-yl)ethyl)indolin-2-one (9c).

Yellow solid, yield: 62%. ^1^H NMR (400 MHz, CDCl_3_) δ 0.73–0.91 (m, 7H), 1.12–1.16 (m, 1H), 1.29–1.34 (m, 1H), 1.48–1.56 (m, 5H), 1.60–1.63 (m, 1H), 1.67–1.70 (m, 2H), 1.76–1.80 (m, 2H), 1.98 (d, *J* = 4.0 Hz, 1H), 2.32–2.34 (m, 1H), 3.50 (d, *J* = 4.0 Hz 1H), 4.14–4.23 (m, 5H), 4.44 (d, *J* = 8.0 Hz, 1H), 4.58–4.63 (m, 3H), 4.70 (d, *J* = 12.0 Hz, 1H), 5.17 (s, 1H), 6.42 (d, *J* = 8.0 Hz, 1H), 6.94 (t, *J* = 8.0 Hz, 1H), 7.20 (t, *J* = 4.0 Hz, 1H), 7.32 (s, 1H), 7.84 (d, *J* = 4.0 Hz, 1H). ^13^C NMR (100 MHz, CDCl_3_) δ 163.84, 145.53, 143.00, 142.87, 132.69, 128.00, 123.43, 123.39, 121.07, 115.47, 108.01, 99.47, 93.57, 84.16, 69.58, 64.96, 61.43, 47.94, 42.54, 40.62, 40.56, 40.53, 34.80, 34.69, 30.33, 30.20, 24.99, 21.02, 18.84, 12.34. HRMS-ESI: m/z Calcd for C_29_H_37_N_5_O_7_Na [M + Na]^+^: 590.2586; Found: 590.2573.

3-(ethoxyimino)-1-(2-(4-((((3*R*,5a*S*,6*R*,8a*S*,9*R*,12*R*,12a*R*)-3,6,9-trimethyldecahydro-12*H*-3,12-epoxy (Willis et al., 2019; Sławiński et al., 2020)dioxepino [4,3-i]isochromen-10-yl)oxy)methyl)-1*H*-1,2,3-triazol-1-yl)ethyl)indolin-2-one (9d).

Yellow solid, yield: 57%. ^1^H NMR (400 MHz, CDCl_3_) δ 0.73–0.91 (m, 7H), 1.13–1.16 (m, 1H), 1.30–1.34 (m, 1H), 1.39 (t, *J* = 4.0 Hz, 3H), 1.48–1.56 (m, 5H), 1.60–1.63 (m, 2H), 1.67–1.70 (m, 1H), 1.76–1.80 (m, 2H), 1.94 (d, *J* = 4.0 Hz, 1H), 2.32–2.34 (m, 1H), 3.50 (d, *J* = 4.0 Hz 1H), 4.20 (q, *J* = 4.0 Hz, 2H), 4.44 (d, *J* = 8.0 Hz, 1H), 4.50 (q, *J* = 4.0 Hz, 2H), 4.60–4.63 (m, 3H), 4.72 (d, *J* = 8.0 Hz, 1H), 5.17 (s, 1H), 6.42 (d, *J* = 4.0 Hz, 1H), 6.95 (t, *J* = 4.0 Hz, 1H), 7.18 (t, *J* = 4.0 Hz, 1H), 7.31 (s, 1H), 7.86 (d, *J* = 4.0 Hz, 1H). ^13^C NMR (100 MHz, CDCl_3_) δ 163.99, 145.54, 142.83, 142.76, 132.51, 127.90, 123.42, 123.35, 115.58, 108.02, 107.94, 99.48, 93.57, 84.17, 73.31, 69.60, 61.44, 48.00, 42.55, 40.63, 40.56, 34.80, 34.70, 30.23, 30.20, 24.99, 21.02, 18.83, 14.70, 12.36. HRMS-ESI: m/z Calcd for C_30_H_39_N_5_O_7_Na [M + Na]^+^: 604.2742; Found: 604.2747.

3-((benzyloxy)imino)-1-(2-(4-((((3*R*,5a*S*,6*R*,8a*S*,9*R*,12*R*,12a*R*)-3,6,9-trimethyldecahydro-12*H*-3,12-epoxy (Willis et al., 2019; Sławiński et al., 2020)dioxepino [4,3-i]isochromen-10-yl)oxy)methyl)-1*H*-1,2,3-triazol-1-yl)ethyl)indolin-2-one (9e).

Yellow solid, yield: 48%. ^1^H NMR (400 MHz, CDCl_3_) δ 0.72–0.88 (m, 7H), 1.12–1.14 (m, 1H), 1.29–1.32 (m, 1H), 1.48–1.56 (m, 5H), 1.59–1.62 (m, 2H), 1.66–1.70 (m, 2H), 1.75–1.79 (m, 2H), 2.33–2.34 (m, 1H), 3.50 (s, 1H), 4.18 (q, *J* = 4.0 Hz, 2H), 4.44 (d, *J* = 12.0 Hz, 1H), 4.63 (td, *J* = 4.0, 2.0 Hz, 2H), 4.72 (d, *J* = 8.0 Hz, 1H), 5.17 (s, 1H), 5.46 (s, 1H), 6.42 (d, *J* = 8.0 Hz, 1H), 6.90 (t, *J* = 8.0 Hz, 1H), 7.16 (t, *J* = 8.0 Hz, 1H), 7.30–7.39 (m, 6H), 7.82 (s, 1H). ^13^C NMR (100 MHz, CDCl_3_) δ 163.89, 145.55, 143.36, 142.88, 135.96, 132.74, 128.68, 128.62, 128.54, 128.15, 123.46, 124.43, 115.53, 108.02, 107.99, 99.50, 93.59, 84.17, 79.66, 69.61, 61.45, 47.98, 42.55, 40.62, 40.58, 34.80, 34.70, 30.33, 30.21, 24.99, 21.02, 18.83, 12.35. HRMS-ESI: m/z Calcd for C_35_H_41_N_5_O_7_Na [M + Na]^+^: 666.2899; Found: 666.2904.

5-methoxy-3-(methoxyimino)-1-(2-(4-((((3*R*,5a*S*,6*R*,8a*S*,9*R*,12*R*,12a*R*)-3,6,9-trimethyldecahydro-12*H*-3,12-epoxy (Willis et al., 2019; Sławiński et al., 2020)dioxepino [4,3-i]isochromen-10-yl)oxy)methyl)-1*H*-1,2,3-triazol-1-yl)ethyl)indolin-2-one (9f).

Yellow solid, yield: 59%. ^1^H NMR (400 MHz, CDCl_3_) δ 0.74–0.91 (m, 7H), 1.11–1.16 (m, 1H), 1.29–1.34 (m, 1H), 1.48–1.55 (m, 5H), 1.60–1.63 (m, 1H), 1.67–1.70 (m, 1H), 1.76–1.80 (m, 2H), 1.98 (d, *J* = 4.0 Hz, 1H), 2.30–2.35 (m, 1H), 3.50 (d, *J* = 4.0 Hz, 1H), 3.69 (s, 3H), 4.12 (q, *J* = 4.0 Hz, 2H), 4.32 (s, 3H), 4.44 (d, *J* = 8.0 Hz, 1H), 4.58 (t, *J* = 4.0 Hz, 2H), 4.64 (d, *J* = 2.0 Hz, 1H), 4.72 (d, *J* = 8.0 Hz, 1H), 5.17 (s, 1H), 6.32 (d, *J* = 4.0 Hz, 1H), 6.72 (dd, *J* = 4.0, 2.0 Hz, 1H), 7.32 (s, 1H), 7.44 (d, *J* = 2.0 Hz, 1H). ^13^C NMR (100 MHz, CDCl_3_) δ 163.75, 156.02, 145.53, 143.28, 136.52, 123.45, 117.32, 116.05, 114.57, 108.54, 108.02, 99.48, 93.60, 84.15, 69.59, 65.01, 61.47, 55.88, 48.00, 42.53, 40.68, 40.62, 34.80, 34.68, 30.33, 30.22, 24.98, 20.99, 18.83, 12.30. HRMS-ESI: m/z Calcd for C_30_H_39_N_5_O_8_Na [M + Na]^+^: 620.2691; Found: 620.2674.

3-(ethoxyimino)-5-methoxy-1-(2-(4-((((3*R*,5a*S*,6*R*,8a*S*,9*R*,12*R*,12a*R*)-3,6,9-trimethyldecahydro-12*H*-3,12-epoxy (Willis et al., 2019; Sławiński et al., 2020)dioxepino [4,3-i]isochromen-10-yl)oxy)methyl)-1*H*-1,2,3-triazol-1-yl)ethyl)indolin-2-one (**9g**).

Yellow solid, yield: 42%. ^1^H NMR (400 MHz, CDCl_3_) δ 0.74–0.92 (m, 7H), 1.13–1.15 (m, 1H), 1.30–1.34 (m, 1H), 1.39 (t, *J* = 4.0 Hz, 3H), 1.48–1.54 (m, 5H), 1.56–1.61 (m, 2H), 1.66–1.69 (m, 1H), 1.76–1.79 (m, 2H), 1.90 (d, *J* = 4.0 Hz, 1H), 2.32–2.35 (m, 1H), 3.51 (d, *J* = 4.0 Hz, 1H), 3.70 (s, 3H), 4.16 (q, *J* = 4.0 Hz, 2H), 4.44 (d, *J* = 8.0 Hz, 1H), 4.50 (q, *J* = 4.0 Hz, 2H), 4.60 (td, *J* = 4.0, 2.0 Hz, 2H), 4.65 (d, *J* = 2.0 Hz, 1H), 4.72 (d, *J* = 8.0 Hz, 1H), 5.17 (s, 1H), 6.30 (d, *J* = 8.0 Hz, 1H), 6.72 (dd, *J* = 4.0, 2.0 Hz, 1H), 7.31 (s, 1H), 7.48 (d, *J* = 4.0 Hz, 1H). ^13^C NMR (100 MHz, CDCl_3_) δ 163.90, 155.99, 145.55, 143.11, 136.42, 123.44, 116.87, 116.21, 114.73, 108.42, 108.02, 99.51, 93.60, 84.17, 73.35, 69.62, 61.49, 55.85, 48.06, 42.55, 40.69, 40.64, 34.80, 34.70, 30.33, 30.23, 24.99, 21.00, 18.83, 14.71, 12.32. HRMS-ESI: m/z Calcd for C_31_H_41_N_5_O_8_Na [M + Na]^+^: 634.2848; Found: 634.2839.

3-[(benzyloxy)imino]-5-methoxy-1-(2-(4-((((3*R*,5a*S*,6*R*,8a*S*,9*R*,12*R*,12a*R*)-3,6,9-trimethyldecahydro-12*H*-3,12-epoxy (Willis et al., 2019; Sławiński et al., 2020)dioxepino [4,3-i]isochromen-10-yl)oxy)methyl)-1*H*-1,2,3-triazol-1-yl)ethyl)indolin-2-one (9h).

Yellow solid, yield: 41%. ^1^H NMR (400 MHz, CDCl_3_) δ 0.73–0.90 (m, 7H), 1.12–1.15 (m, 1H), 1.28–1.33 (m, 1H), 1.48–1.56 (m, 5H), 1.60–1.62 (m, 2H), 1.66–1.69 (m, 1H), 1.76–1.79 (m, 2H), 1.90 (d, *J* = 8.0 Hz, 1H), 2.31–2.34 (m, 1H), 3.50 (d, *J* = 4.0 Hz, 1H), 3.62 (s, 1H), 4.16 (q, *J* = 4.0 Hz, 2H), 4.44 (d, *J* = 8.0 Hz, 1H), 4.58 (td, *J* = 4.0, 2.0 Hz, 2H), 4.66 (d, *J* = 4.0 Hz, 1H), 4.72 (d, *J* = 8.0 Hz, 1H), 5.17 (s, 1H), 5.46 (s, 1H), 6.30 (d, *J* = 4.0 Hz, 1H), 6.70 (dd, *J* = 4.0, 2.0 Hz, 1H), 7.28–7.39 (m, 6H), 7.44 (d, *J* = 2.0 Hz, 1H). ^13^C NMR (100 MHz, CDCl_3_) δ 163.79, 156.01, 145.56, 143.70, 136.54, 135.97, 128.67, 128.61, 128.44, 123.45, 117.25, 116.13, 114.78, 108.52, 108.02, 99.51, 93.62, 84.16, 79.61, 69.62, 61.49, 55.77, 48.04, 42.54, 40.71, 40.63, 34.80, 34.69, 20.33, 30.23, 24.98, 21.00, 18.83, 12.32. HRMS-ESI: m/z Calcd for C_36_H_43_N_5_O_8_Na [M + Na]^+^: 696.3004; Found: 696.2981.

5-fluoro-3-(hydroxyimino)-1-(2-(4-((((3*R*,5a*S*,6*R*,8a*S*,9*R*,12*R*,12a*R*)-3,6,9-trimethyldecahydro-12*H*-3,12-epoxy (Willis et al., 2019; Sławiński et al., 2020)dioxepino [4,3-i]isochromen-10-yl)oxy)methyl)-1*H*-1,2,3-triazol-1-yl)ethyl)indolin-2-one (9i).

Yellow solid, yield: 67%. ^1^H NMR (400 MHz, CD_3_OD) δ 0.72–0.88 (m, 7H), 1.11–1.13 (m, 1H), 1.19–1.21 (m, 1H), 1.37–1.41 (m, 4H), 1.48–1.59 (m, 3H), 1.64–1.69 (m, 2H), 1.75–1.78 (m, 1H), 2.28–2.30 (m, 1H), 3.39 (d, *J* = 2.0 Hz, 1H), 4.16 (t, *J* = 4.0 Hz, 1H), 4.40 (d, *J* = 8.0 Hz, 1H), 4.60–4.67 (m, 4H), 5.16 (s, 1H), 6.64 (dd, *J* = 4.0, 2.0 Hz, 1H), 6.98 (td, *J* = 8.0, 2.0 Hz, 1H), 7.66 (dd, *J* = 8.0, 2.0 Hz, 1H), 7.80 (s, 1H). ^13^C NMR (100 MHz, CD_3_OD) δ 164.50, 159.72 (*J* = 198.75 Hz), 144.84, 142.97, 138.62, 124.46, 117.69, 117.53, 116.17, 114.39, 114.21, 109.14, 109.09, 107.92, 98.97, 93.51, 83.73, 68.64, 60.32, 42.10, 40.67, 40.03, 34.52, 30.24, 30.13, 24.80, 20.02, 17.88, 11.28. HRMS-ESI: m/z Calcd for C_28_H_34_FN_5_O_7_Na [M + Na]^+^: 594.2335; Found: 594.2330.

5-fluoro-3-(methoxyimino)-1-(2-(4-((((3*R*,5a*S*,6*R*,8a*S*,9*R*,12*R*,12a*R*)-3,6,9-trimethyldecahydro-12*H*-3,12-epoxy (Willis et al., 2019; Sławiński et al., 2020)dioxepino [4,3-i]isochromen-10-yl)oxy)methyl)-1*H*-1,2,3-triazol-1-yl)ethyl)indolin-2-one (9j).

Yellow solid, yield: 52%. ^1^H NMR (400 MHz, CDCl_3_) δ 0.74–0.90 (m, 7H), 1.13–1.15 (m, 1H), 1.30–1.33 (m, 1H), 1.48–1.56 (m, 6H), 1.60–1.63 (m, 1H), 1.68–1.71 (m, 1H), 1.76–1.80 (m, 2H), 1.88 (d, *J* = 8.0 Hz, 1H), 2.33–2.35 (m, 1H), 3.50 (dd, *J* = 8.0, 4.0 Hz, 1H), 4.15–4.25 (m, 4H), 4.44 (d, *J* = 8.0 Hz, 1H), 4.60 (t, *J* = 4.0 Hz, 1H), 4.65 (d, *J* = 4.0 Hz, 1H), 4.74 (d, *J* = 8.0 Hz, 1H), 5.18 (s, 1H), 6.34 (dd, *J* = 4.0, 2.0 Hz, 1H), 6.90 (t, *J* = 4.0 Hz, 1H), 7.30 (s, 1H), 7.58 (dd, *J* = 4.0, 2.0 Hz, 1H). ^13^C NMR (100 MHz, CDCl_3_) δ 174.32, 159.74 (*J* = 198.75 Hz), 145.63, 123.46, 118.98, 118.54, 108.03, 99.48, 93.95, 84.15, 69.63, 65.25, 61.48, 48.00, 42.52, 40.78, 40.62, 34.82, 30.32, 30.22, 24.98, 20.98, 18.83, 12.27. HRMS-ESI: m/z Calcd for C_29_H_36_FN_5_O_7_Na [M + Na]^+^: 608.2491; Found: 608.2483.

3-(ethoxyimino)-5-fluoro-1-(2-(4-((((3*R*,5a*S*,6*R*,8a*S*,9*R*,12*R*,12a*R*)-3,6,9-trimethyldecahydro-12*H*-3,12-epoxy (Willis et al., 2019; Sławiński et al., 2020)dioxepino [4,3-i]isochromen-10-yl)oxy)methyl)-1*H*-1,2,3-triazol-1-yl)ethyl)indolin-2-one (9k).

Yellow solid, yield: 33%. ^1^H NMR (400 MHz, CDCl_3_) δ 0.73–0.90 (m, 7H), 1.16–1.18 (m, 1H), 1.32–1.33 (m, 1H), 1.40 (t, *J* = 4.0 Hz, 3H), 1.48–1.56 (m, 5H), 1.60–1.70 (m, 3H), 1.76–1.80 (m, 2H), 1.92 (d, *J* = 8.0 Hz, 1H), 2.33–2.35 (m, 1H), 3.49–3.51 (m, 1H), 4.17–4.20 (m, 2H), 4.44 (d, *J* = 8.0 Hz, 1H), 4.50 (q, *J* = 4.0 Hz, 2H), 4.60 (t, *J* = 4.0 Hz, 1H), 4.66 (d, *J* = 4.0 Hz, 1H), 4.74 (d, *J* = 8.0 Hz, 1H), 5.18 (s, 1H), 6.34 (dd, *J* = 4.0, 2.0 Hz, 1H), 6.88 (t, *J* = 4.0 Hz, 1H), 7.31 (s, 1H), 7.60 (dd, *J* = 4.0, 2.0 Hz, 1H). ^13^C NMR (100 MHz, CDCl_3_) δ 163.77, 159.68 (*J* = 200.00 Hz), 145.60, 142.44, 138.82, 123.49, 118.83, 118.67, 116.03, 115.43, 115.26, 108.72, 108.66, 108.03, 99.46, 93.64, 84.14, 73.69, 69.61, 61.46, 48.05, 42.51, 40.77, 40.61, 34.82, 34.68, 30.33, 30.22, 24.98, 20.98, 18.82, 14.69, 12.28. HRMS-ESI: m/z Calcd for C_30_H_38_FN_5_O_7_Na [M + Na]^+^: 622.2648; Found: 622.2630.

### Antiproliferative Activity

A549, A549/DOX, and A549/DDP lung cancer cells (2 × 10^3^) were plated in each well of a 96-well plate and were allowed to adhere and spread for 24 h. The 1,2,3-triazole tethered dihydroartemisinin-isatin hybrids 8a-c and 9a-k were added to a final concentration of 100 μM, and the cells were cultured for 24 h at 37°C. 3-(4,5-dimethyl-2-thiazolyl)-2,5-diphenyltetrazolium bromide (MTT) solution (10 *µ*L) was added to each well, and the cultures were incubated for an additional 4 h. A further 100 *µ*L of MTT solution was added and incubation continued overnight. The absorbance at 540 nm was determined in each well with a 96-well plate reader. The growth of the treated cells was compared with that of untreated cells.

### Cytotoxicity

The cytotoxicity (CC_50_) of the synthesized 1,2,3-triazole tethered dihydroartemisinin-isatin hybrids 8a-c and 9a-k were examined by the MTT assay in mouse embryonic fibroblast cells NIH/3T3. The compounds were dissolved in DMSO with concentrations from 1,024 to 1 μg/ml. The NIH/3T3 cells were maintained in culture medium at 37°C under 5% CO_2_ atmosphere. Cells were seeded in 96-well plates (1 × 10^4^ cell per well) and allowed to recover for 24 h. After 72 h of exposure, cells were harvested and cell viability was assessed by MTT assay. The CC_50_ values were calculated by Bliss analy.

### Pharmacokinetic Profiles Determination

CD-1 mice (20–25 g) were used in the pharmacokinetic study, and each treatment group had 3 mice which were dosed with hybrids 8a,c suspension at 30 mg/kg by single intravenous administration. Compounds were suspended in 0.5% CMC for iv, and blood was collected from the jugular vein of each mouse at the following time points: 0.25, 0.5, 1, 2, 4, 6, 8 and 24 h after intravenous administration. Total area under the concentration time curve (AUC), the elimination half-time (t_1/2_), the peak concentration (C_max_) and the time to reach peak concentration (T_max_) of samples were determined directly from the experimental data using WinNonlin V6.2.1.

## Data Availability

The original contributions presented in the study are included in the article/Supplementary Material, further inquiries can be directed to the corresponding author.
