# Supplementary material for: Design, Synthesis and Anti-Lung Cancer Evaluation of 1, 2, 3-Triazole Tethered Dihydroartemisinin-Isatin Hybrids
Source: Front Pharmacol. 2021 Dec 16;12:801580. doi: 10.3389/fphar.2021.801580 (PMC8716824; doi:10.3389/fphar.2021.801580)

**Design, synthesis and anti-lung cancer evaluation of 1,2,3-triazole tethered dihydroartemisinin-isatin hybrids**

Haodong Hou, Bin Qu, Chen Su, Guihua Hou, Feng Gao^*^

Key Laboratory for Experimental Teratology of the Ministry of Education and Center for Experimental Nuclear Medicine, School of Basic Medical Sciences, Cheeloo College of Medicine, Shandong University, Jinan, Shandong, 250012, China

1. **Materials**

^1^H NMR and ^13^C NMR spectra were determined on a Varian Mercury-400 spectrometer in CDCl^3^ using tetramethylsilane (TMS) as an internal standard. Electrospray ionization (ESI) mass spectra were obtained on a MDSSCIEXQ-Tap mass spectrometer. Unless otherwise noted, the reagents were obtained from commercial supplier and were used without further purification. A549, A549/DOX, and A549/DDP lung cancer cell lines were purchased from the American Type Culture Collection (ATCC) and preserved by Center for Experimental Nuclear Medicine of Shandong University.

1. **Synthesis**

To a mixture of dihydroartemisinin **1** (100 mmol) and propargyl alcohol **2** (120 mmol) in DCM (500 mL) was added boron trifluoride diethyl etherate (BF_3_**^.^**OEt_2_, 20 mL) at 0 ^o^C, and the mixture was stirred at room temperatire overnight. Sat. Na_2_CO_3_ (500 mL) was added to the mixture, and then the organic layer was separated. The organic layer was washed with H_2_O (500 mL) and brine (500 mL) in sequence, dried over anhydrous Na_2_SO_4_, filterated, and concentrated under reduced pressure to give crude alkynyl-containing dihydroartemisinin intermediate **3**.

To a solution of isatins **4** (100 mmol) in DMF (100 mL), potassium carbonate (K_2_CO_3_, 200 mmol) was added. The mixture was stirred at room temperature for 1 h, and then 1,2-dibromoethane (**5**, 150 mmol) was added. The mixture was stirred overnight at room temperature, and then filtered. The mixture was concentrated under reduced pressure and the residue was purified by silica gel chromatography eluted with PE to PE:EA=2:1 to provide intermediates **6**.

A mixture of intermediates **6** (10 mmol) and NaN_3_ (15 mmol) in DMF (30 mmol) was stirred at 50 ^o^C for 12 h, and then cooled to room temperature. H_2_O (100 mL) was added to the mixture, and the mixture was extracted with DCM (100 mL*3). The combined organic layers were washed with H_2_O (500 mL) and brine (500 mL) in sequence, dried over anhydrous Na_2_SO_4_, filterated, and concentrated under reduced pressure to give crude azido precursors **7**.

The mixture of intermediates **6** (3 mmol), precursors **7** (3 mmol) and CuSO_4_ (1 mmol) in DMF (10 mmol) was stirred at 60 ^o^C for 8 h under N_2_ atmosphere, and then cooled to room temperature. After filteration, the filtrate was concentrated under reduced pressure. The residue was purified by silica gel chromatography eluted with PE to PE:EA=1:2 to generate 1,2,3-triazole tethered dihydroartemisinin-isatin hybrids **8a-c**.

To a solution of hybrids **8a-c** (1 mmol) and amine hydrochlorides (1.5 mmol) in a mixture of EtOH (10 mL) and H_2_O (10 mL), Na_2_CO_3_ (2 mmol) was added. The mixture was stirred at 60 ^o^C for 12 h, and then cooled to room temperature. The mixture was extracted with DCM (20 mL*3). The combined organic layers were washed with H_2_O (30 mL) and brine (30 mL) in sequence, dried over anhydrous Na_2_SO_4_, filterated, and concentrated under reduced pressure. The residue was purified by silica gel chromatography eluted with PE to PE:EA=1:2 to give 1,2,3-triazole tethered dihydroartemisinin-isatin hybrids **9a-k**.

1-(2-(4-((((3*R*,5a*S*,6*R*,8a*S*,9*R*,12*R*,12a*R*)-3,6,9-trimethyldecahydro-12*H*-3,12-epoxy[1,2]dioxepino[4,3-i]isochromen-10-yl)oxy)methyl)-1*H*-1,2,3-triazol-1-yl)ethyl)indoline-2,3-dione (**8a**)

Red solid, yield: 37%. ^1^H NMR (400 MHz, CDCl_3_) δ 0.75-0.92 (m, 7H), 1.11-1.14 (m, 1H), 1.31-1.35 (m, 1H), 1.48-1.55 (m, 5H), 1.60-1.63 (m, 1H), 1.68-1.71 (m, 2H), 1.76-1.80 (m, 2H), 1.99-2.06 (m, 1H), 2.33-2.36 (m, 1H), 3.50 (d, *J* = 4.0 Hz, 1H), 4.18 (t, *J* = 4.0 Hz, 2H), 4.46 (d, *J* = 8.0 Hz, 1H), 4.69 (t, *J* = 4.0 Hz, 2H), 4.74 (dd, *J* = 8.0, 4.0 Hz, 1H), 5.18 (s, 1H), 6.52 (d, *J* = 4.0 Hz, 1H), 7.00 (d, *J* = 4.0 Hz, 1H), 7.42 (t, *J* = 4.0 Hz, 1H), 7.50 (d, *J* = 4.0 Hz, 1H). ^13^C NMR (100 MHz, CDCl_3_) δ 182.36, 158.57, 150.05, 145.72, 138.69, 125.61, 124.20, 123.54, 117.38, 109.63, 108.02, 99.51, 93.67, 84.12, 69.56, 61.51, 47.70, 42.47, 40.73, 40.59, 34.83, 34.67, 30.33, 30.23, 25.00, 21.00, 18.82, 12.31. HRMS-ESI: m/z Calcd for C_28_H_34_N_4_O_7_Na [M+Na]^+^: 561.2320; Found: 561.2314.


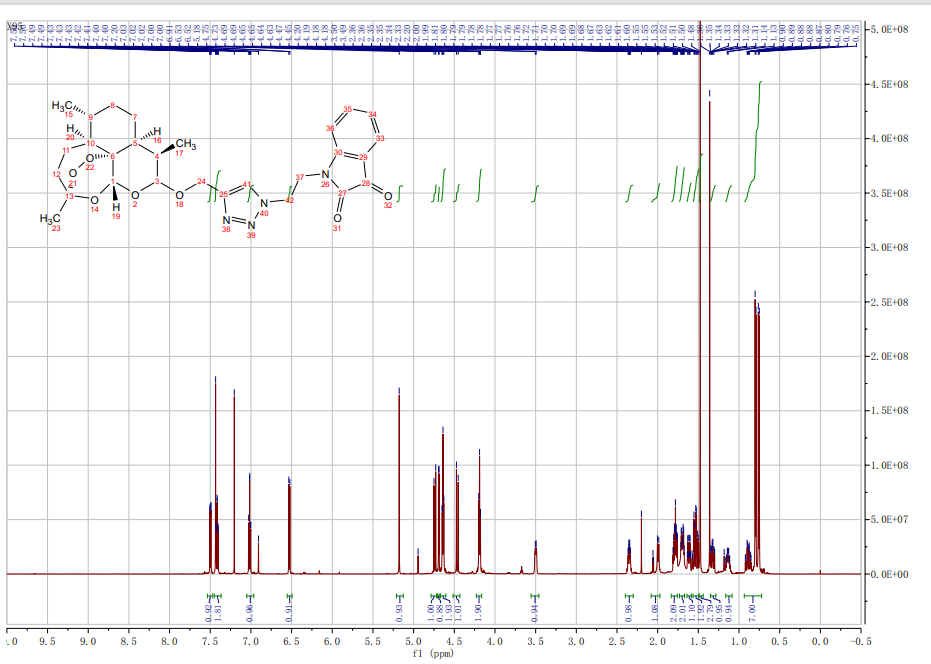


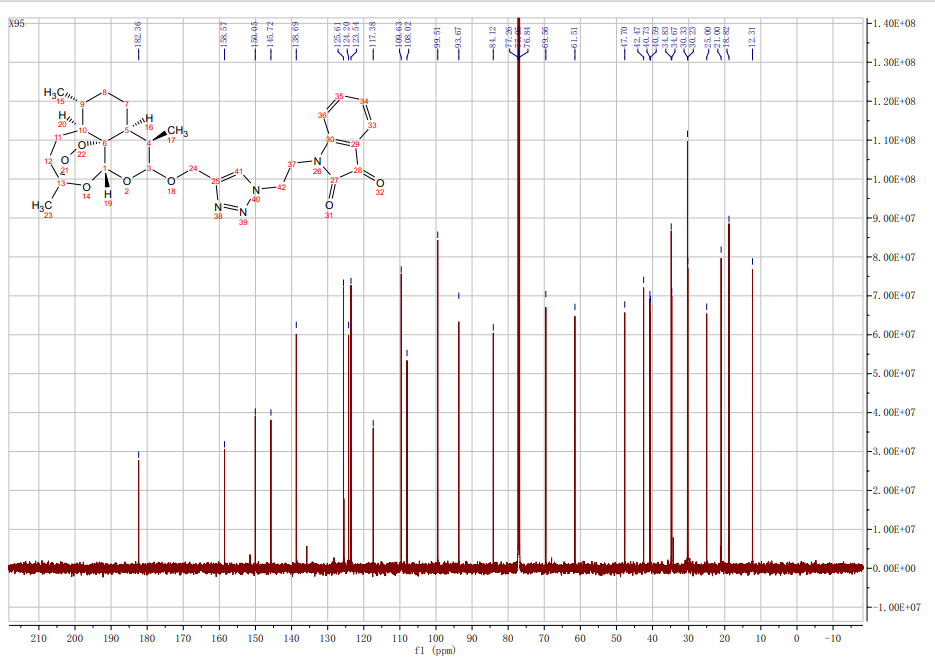


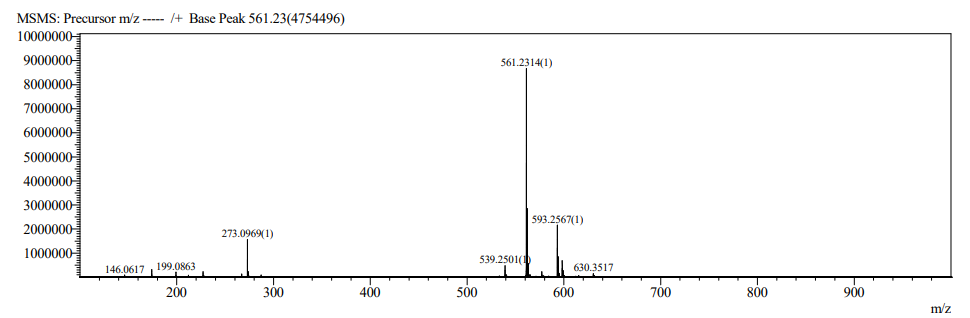


5-methoxy-1-(2-(4-((((3*R*,5a*S*,6*R*,8a*S*,9*R*,12*R*,12a*R*)-3,6,9-trimethyldecahydro-12*H*-3,12-epoxy[1,2]dioxepino[4,3-i]isochromen-10-yl)oxy)methyl)-1*H*-1,2,3-triazol-1-yl)ethyl)indoline-2,3-dione (**8b**)

Red solid, yield: 28%. ^1^H NMR (400 MHz, CDCl_3_) δ 0.75-0.92 (m, 7H), 1.13-1.17 (m, 1H), 1.30-1.35 (m, 1H), 1.48-1.56 (m, 5H), 1.60-1.63 (m, 1H), 1.68-1.70 (m, 2H), 1.76-1.81 (m, 2H), 1.98 (d, *J* = 8.0 Hz, 1H), 2.34-2.36 (m, 1H), 3.50 (d, *J* = 4.0 Hz, 1H), 3.70 (s, 3H), 4.15 (t, *J* = 4.0 Hz, 2H), 4.48 (d, *J* = 12.0 Hz, 1H), 4.62 (t, *J* = 4.0 Hz, 2H), 4.70 (dd, *J* = 2.0 Hz, 1H), 4.74 (d, *J* = 4.0 Hz, 1H), 5.18 (s, 1H), 6.44 (d, *J* = 4.0 Hz, 1H), 6.94 (dd, *J* = 4.0, 2.0 Hz, 1H), 7.02 (d, *J* = 4.0 Hz, 1H), 7.20 (s, 1H), 7.93 (s, 1H). ^13^C NMR (100 MHz, CDCl_3_) δ 182.72, 158.70, 156.70, 145.72, 143.88, 135.79, 125.52, 124.80, 123.53, 117.84, 110.68, 109.80, 108.02, 99.49, 93.70, 84.12, 69.58, 61.52, 55.93, 47.78, 42.47, 40.78, 40.61, 34.83, 34.68, 30.33, 30.25, 24.99, 20.99, 18.82, 12.27. HRMS-ESI: m/z Calcd for C_29_H_36_N_4_O_8_Na [M+Na]^+^: 591.2426; Found: 591.2410.


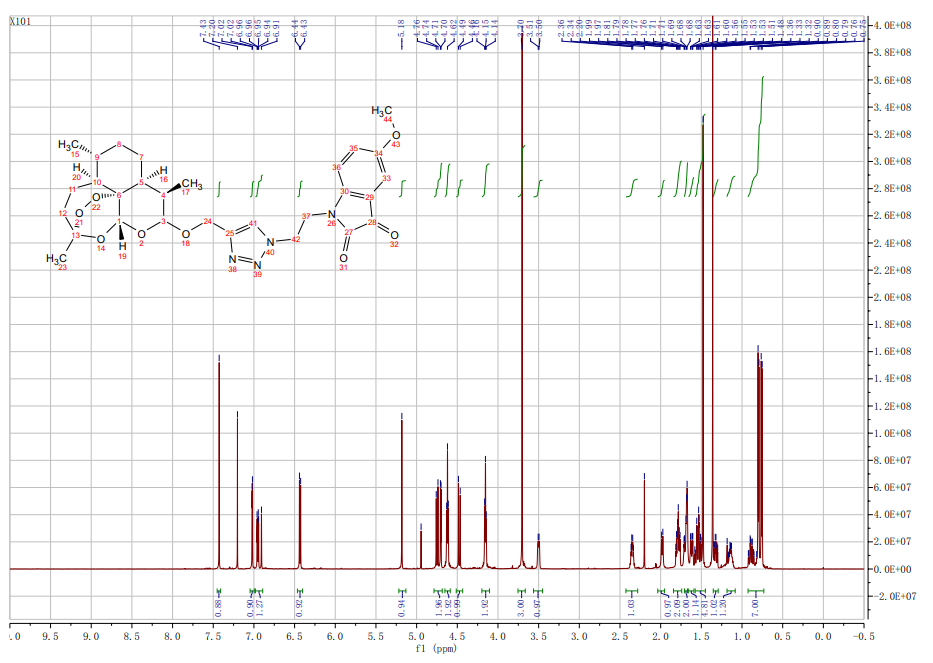


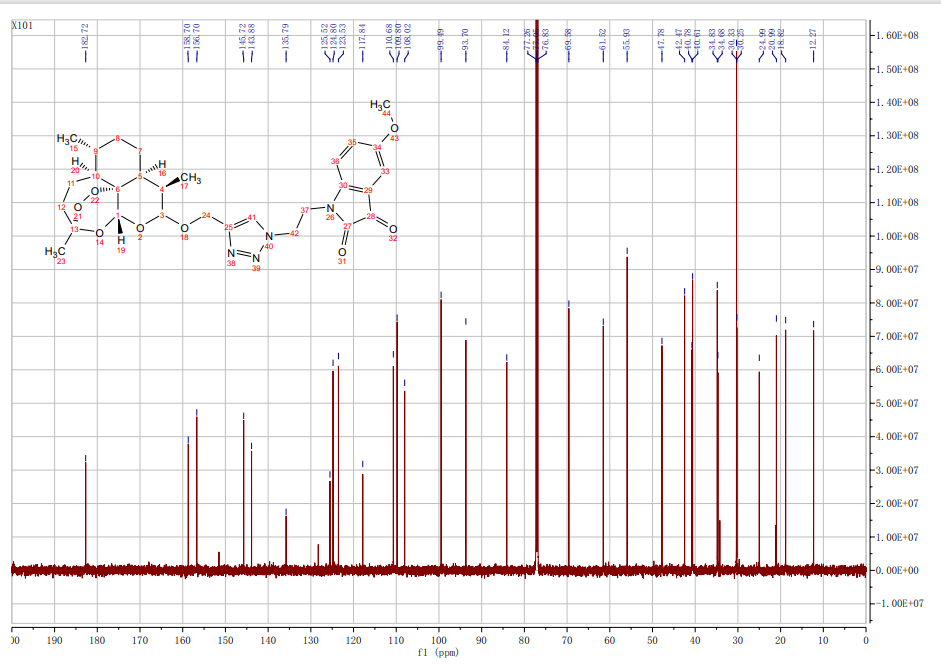


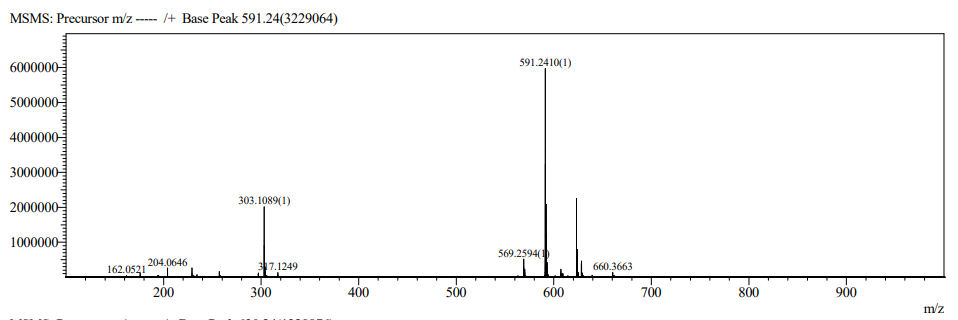


5-fluoro-1-(2-(4-((((3*R*,5a*S*,6*R*,8a*S*,9*R*,12*R*,12a*R*)-3,6,9-trimethyldecahydro-12*H*-3,12-epoxy[1,2]dioxepino[4,3-i]isochromen-10-yl)oxy)methyl)-1*H*-1,2,3-triazol-1-yl)ethyl)indoline-2,3-dione (**8c**)

Red solid, yield: 33%. ^1^H NMR (400 MHz, CDCl_3_) δ 0.76-0.93 (m, 7H), 1.13-1.14 (m, 1H), 1.33-1.35 (m, 1H), 1.48-1.56 (m, 5H), 1.60-1.63 (m, 1H), 1.68-1.71 (m, 3H), 1.76-1.82 (m, 2H), 1.98-2.06 (m, 1H), 2.34-2.37 (m, 1H), 3.50 (s, 1H), 4.20 (t, *J* = 4.0 Hz, 2H), 4.46 (d, *J* = 8.0 Hz, 1H), 4.63 (t, *J* = 4.0 Hz, 2H), 4.80 (dd, *J* = 8.0, 4.0 Hz, 1H), 5.18 (s, 1H), 6.50 (dd, *J* = 8.0, 4.0 Hz, 1H), 7.12 (td, *J* = 8.0, 2.0 Hz, 1H), 7.20 (dd, *J* = 4.0, 2.0 Hz, 1H), 7.44 (s, 1H). ^13^C NMR (100 MHz, CDCl_3_) δ 181.80, 160.19 (*J* = 205.00 Hz), 158.35, 146.13, 145.79, 127.10, 124.94, 123.63, 118.01, 117.97, 112.65, 112.49, 111.03, 110.98, 108.03, 99.47, 93.75, 84.08, 69.56, 61.53, 47.74, 42.42, 40.88, 40.57, 34.84, 34.65, 30.32, 30.24, 24.99, 20.97, 18.81, 12.22. HRMS-ESI: m/z Calcd for C_28_H_33_FN_4_O_7_Na [M+Na]^+^: 579.2226; Found: 579.2233.


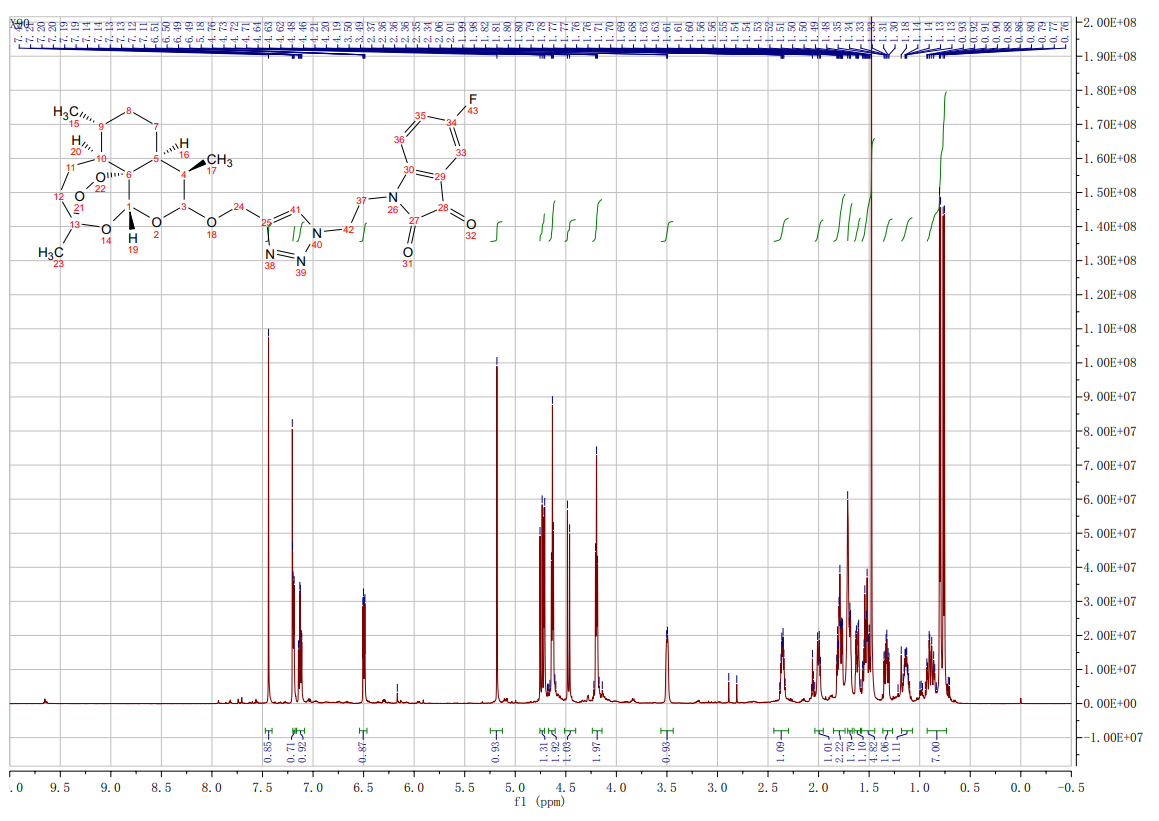


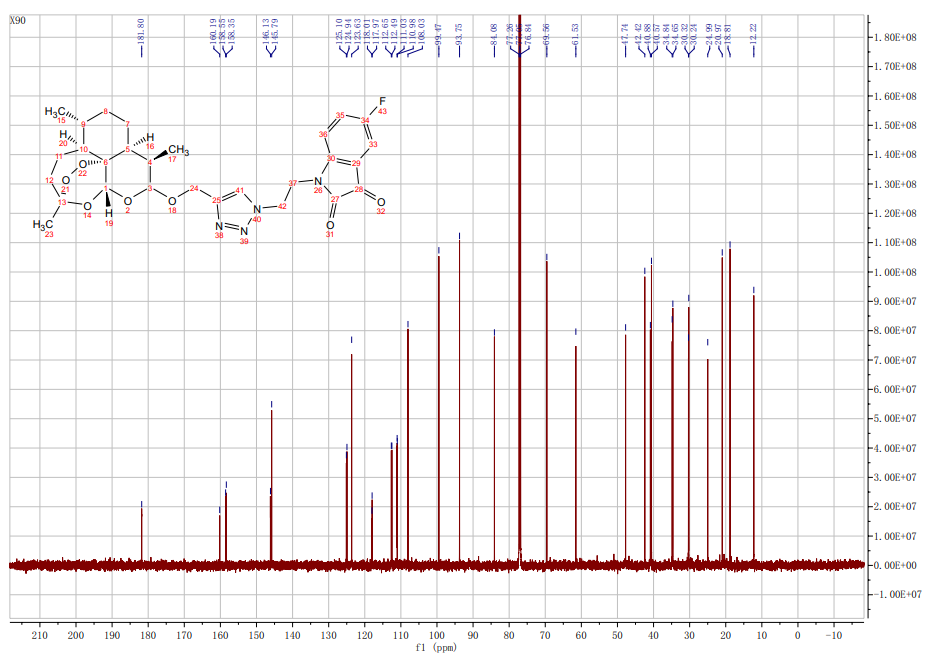


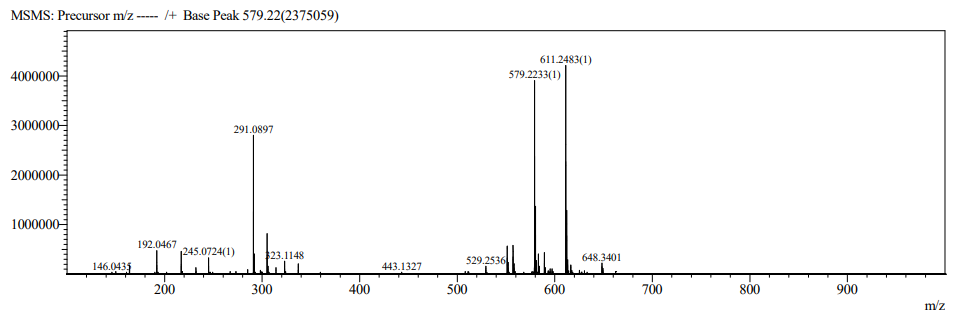


2-(2-oxo-1-(2-(4-((((3*R*,5a*S*,6*R*,8a*S*,9*R*,12*R*,12a*R*)-3,6,9-trimethyldecahydro-12*H*-3,12-epoxy[1,2]dioxepino[4,3-i]isochromen-10-yl)oxy)methyl)-1*H*-1,2,3-triazol-1-yl)ethyl)indolin-3-ylidene)hydrazine-1-carbothioamide (**9a**)

Yellow solid, yield: 49%. ^1^H NMR (400 MHz, CDCl_3_) δ 0.75-0.92 (m, 7H), 1.12-1.16 (m, 1H), 1.31-1.36 (m, 1H), 1.48-1.56 (m, 5H), 1.60-1.62 (m, 1H), 1.69-1.72 (m, 2H), 1.72-1.81 (m, 2H), 2.05-2.06 (m, 1H), 2.33-2.36 (m, 1H), 3.50 (d, , *J* = 2.0 Hz, 1H), 4.20 (td, *J* = 4.0, 2.0 Hz, 2H), 4.46 (d, *J* = 8.0 Hz, 1H), 4.61-4.65 (m, 2H), 4.67 (d, *J* = 2.0 Hz, 1H), 4.72 (d, *J* = 8.0, 4.0 Hz, 1H), 5.18 (s, 1H), 6.52 (d, *J* = 4.0 Hz, 1H), 6.75 (s, 1H), 7.00 (t, *J* = 4.0 Hz, 1H), 7.22 (t, *J* = 4.0 Hz, 1H), 7.38 (s, 1H), 7.46 (d, *J* = 4.0 Hz, 1H), 7.49 (s, 1H), 12.60 (s, 1H). ^13^C NMR (100 MHz, CDCl_3_) δ 179.92, 161.24, 145.64, 142.37, 131.76, 131.15, 123.69, 123.37, 121.01, 119.06, 108.84, 108.02, 99.32, 93.71, 84.13, 69.58, 61.40, 47.72, 42.45, 40.59, 40.36, 34.83, 34.70, 30.34, 30.25, 25.01, 21.01, 18.84, 12.31. HRMS-ESI: m/z Calcd for C_29_H_37_N_7_O_6_SNa [M+Na]^+^: 634.2419; Found: 634.2407.


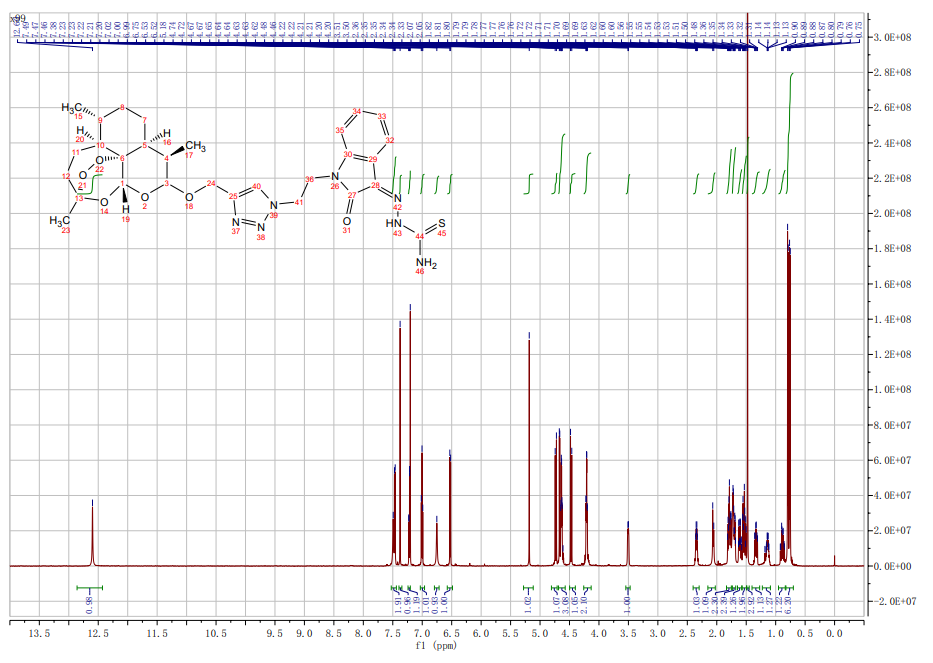


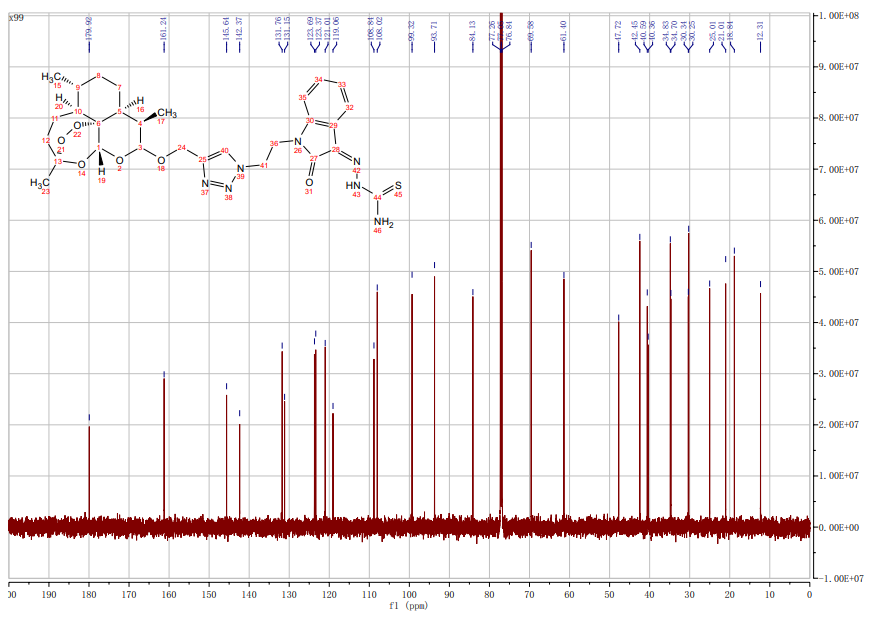


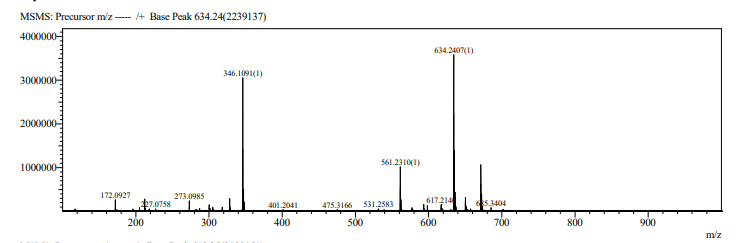


3-(hydroxyimino)-1-(2-(4-((((3*R*,5a*S*,6*R*,8a*S*,9*R*,12*R*,12a*R*)-3,6,9-trimethyldecahydro-12*H*-3,12-epoxy[1,2]dioxepino[4,3-i]isochromen-10-yl)oxy)methyl)-1*H*-1,2,3-triazol-1-yl)ethyl)indolin-2-one (**9b**)

Yellow solid, yield: 83%. ^1^H NMR (400 MHz, CD_3_OD) δ 0.71-0.90 (m, 7H), 1.07-1.20 (m, 2H), 1.36-1.41 (m, 4H), 1.49-1.56 (m, 3H), 1.67-1.70 (m, 2H), 1.74-1.78 (m, 1H), 2.26-2.29 (m, 1H), 3.40 (d, *J* = 2.0 Hz, 1H), 4.16 (t, *J* = 4.0 Hz, 1H), 4.40 (d, *J* = 8.0 Hz, 1H), 4.57-4.68 (m, 4H), 5.15 (s, 1H), 6.66 (d, *J* = 4.0 Hz, 1H), 6.96 (t, *J* = 8.0 Hz, 1H), 7.22 (d, *J* = 8.0 Hz, 1H), 7.78 (s, 1H), 7.92 (d, *J* = 4.0 Hz, 1H). ^13^C NMR (100 MHz, CD_3_OD) δ 164.80, 144.81, 143.34, 142.37, 131.67, 127.21, 124.47, 123.04, 115.55, 108.17, 107.99, 98.98, 93.50, 83.80, 68.59, 60.31, 42.08, 40.59, 39.92, 34.53, 34.49, 30.20, 30.08, 24.81, 20.09, 17.92, 11.34. HRMS-ESI: m/z Calcd for C_30_H_39_FN_5_O_7_Na [M+Na]^+^: 576.2429; Found: 576.2440.


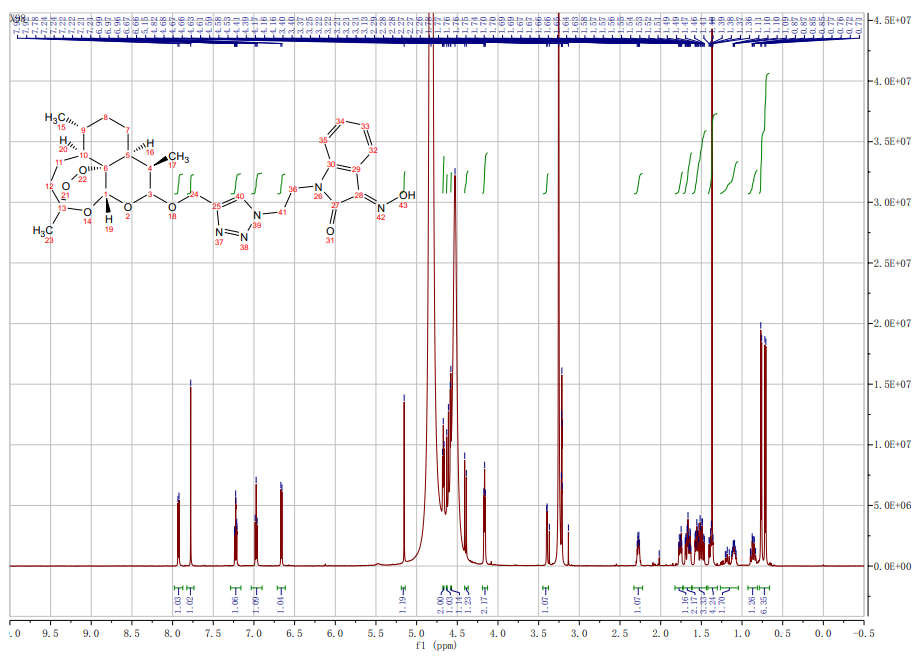


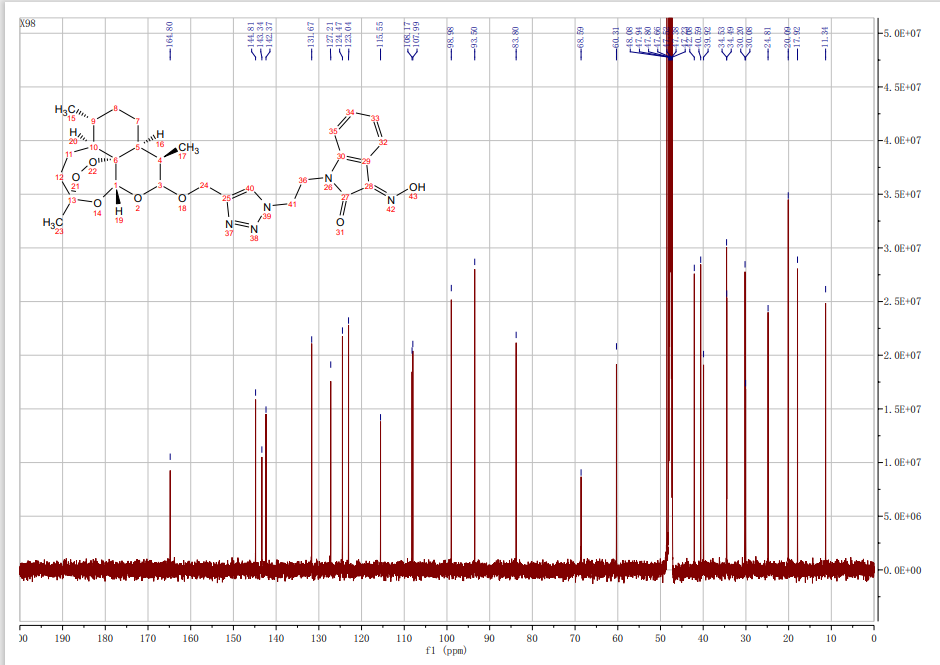


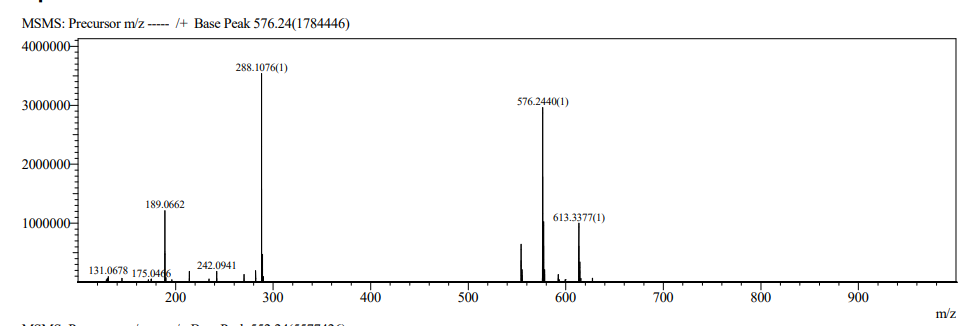


3-(methoxyimino)-1-(2-(4-((((3*R*,5a*S*,6*R*,8a*S*,9*R*,12*R*,12a*R*)-3,6,9-trimethyldecahydro-12*H*-3,12-epoxy[1,2]dioxepino[4,3-i]isochromen-10-yl)oxy)methyl)-1*H*-1,2,3-triazol-1-yl)ethyl)indolin-2-one (**9c**)

Yellow solid, yield: 62%. ^1^H NMR (400 MHz, CDCl_3_) δ 0.73-0.91 (m, 7H), 1.12-1.16 (m, 1H), 1.29-1.34 (m, 1H), 1.48-1.56 (m, 5H), 1.60-1.63 (m, 1H), 1.67-1.70 (m, 2H), 1.76-1.80 (m, 2H), 1.98 (d, *J* = 4.0 Hz, 1H), 2.32-2.34 (m, 1H), 3.50 (d, *J* = 4.0 Hz 1H), 4.14-4.23 (m, 5H), 4.44 (d, *J* = 8.0 Hz, 1H), 4.58-4.63 (m, 3H), 4.70 (d, *J* = 12.0 Hz, 1H), 5.17 (s, 1H), 6.42 (d, *J* = 8.0 Hz, 1H), 6.94 (t, *J* = 8.0 Hz, 1H), 7.20 (t, *J* = 4.0 Hz, 1H), 7.32 (s, 1H), 7.84 (d, *J* = 4.0 Hz, 1H). ^13^C NMR (100 MHz, CDCl_3_) δ 163.84, 145.53, 143.00, 142.87, 132.69, 128.00, 123.43, 123.39, 121.07, 115.47, 108.01, 99.47, 93.57, 84.16, 69.58, 64.96, 61.43, 47.94, 42.54, 40.62, 40.56, 40.53, 34.80, 34.69, 30.33, 30.20, 24.99, 21.02, 18.84, 12.34. HRMS-ESI: m/z Calcd for C_29_H_37_N_5_O_7_Na [M+Na]^+^: 590.2586; Found: 590.2573.


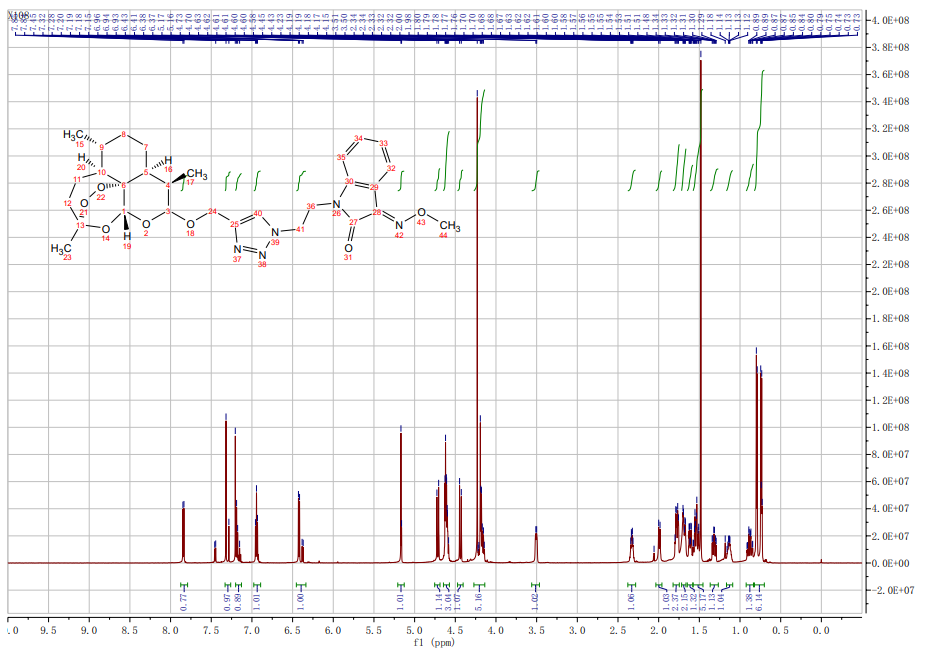


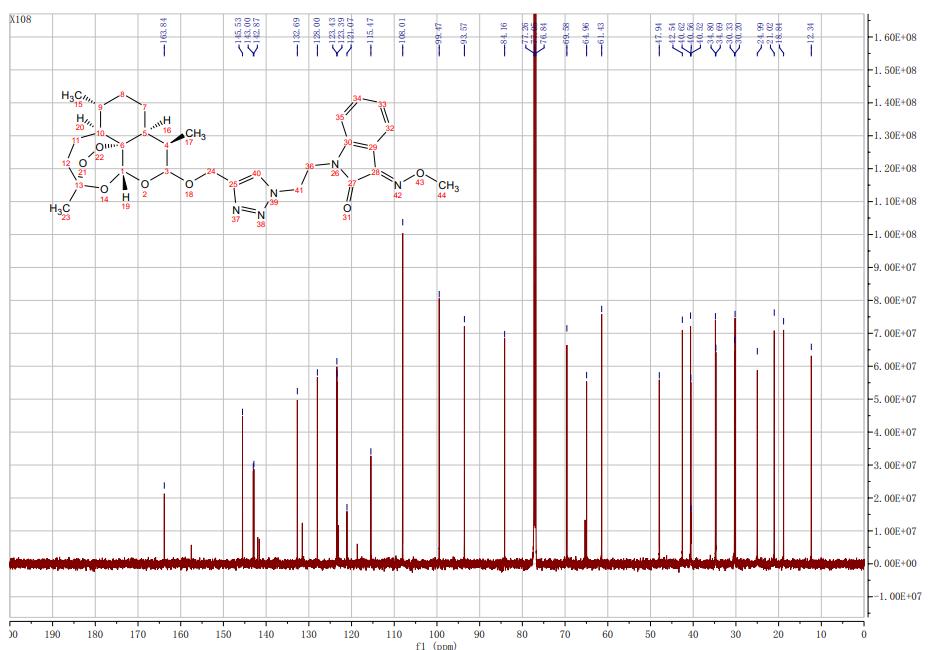


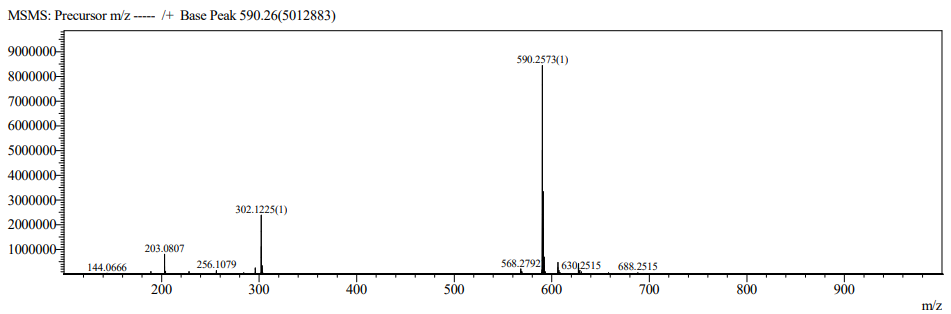


3-(ethoxyimino)-1-(2-(4-((((3*R*,5a*S*,6*R*,8a*S*,9*R*,12*R*,12a*R*)-3,6,9-trimethyldecahydro-12*H*-3,12-epoxy[1,2]dioxepino[4,3-i]isochromen-10-yl)oxy)methyl)-1*H*-1,2,3-triazol-1-yl)ethyl)indolin-2-one (**9d**)

Yellow solid, yield: 57%. ^1^H NMR (400 MHz, CDCl_3_) δ 0.73-0.91 (m, 7H), 1.13-1.16 (m, 1H), 1.30-1.34 (m, 1H), 1.39 (t, *J* = 4.0 Hz, 3H), 1.48-1.56 (m, 5H), 1.60-1.63 (m, 2H), 1.67-1.70 (m, 1H), 1.76-1.80 (m, 2H), 1.94 (d, *J* = 4.0 Hz, 1H), 2.32-2.34 (m, 1H), 3.50 (d, *J* = 4.0 Hz 1H), 4.20 (q, *J* = 4.0 Hz, 2H), 4.44 (d, *J* = 8.0 Hz, 1H), 4.50 (q, *J* = 4.0 Hz, 2H), 4.60-4.63 (m, 3H), 4.72 (d, *J* = 8.0 Hz, 1H), 5.17 (s, 1H), 6.42 (d, *J* = 4.0 Hz, 1H), 6.95 (t, *J* = 4.0 Hz, 1H), 7.18 (t, *J* = 4.0 Hz, 1H), 7.31 (s, 1H), 7.86 (d, *J* = 4.0 Hz, 1H). ^13^C NMR (100 MHz, CDCl_3_) δ 163.99, 145.54, 142.83, 142.76, 132.51, 127.90, 123.42, 123.35, 115.58, 108.02, 107.94, 99.48, 93.57, 84.17, 73.31, 69.60, 61.44, 48.00, 42.55, 40.63, 40.56, 34.80, 34.70, 30.23, 30.20, 24.99, 21.02, 18.83, 14.70, 12.36. HRMS-ESI: m/z Calcd for C_30_H_39_N_5_O_7_Na [M+Na]^+^: 604.2742; Found: 604.2747.


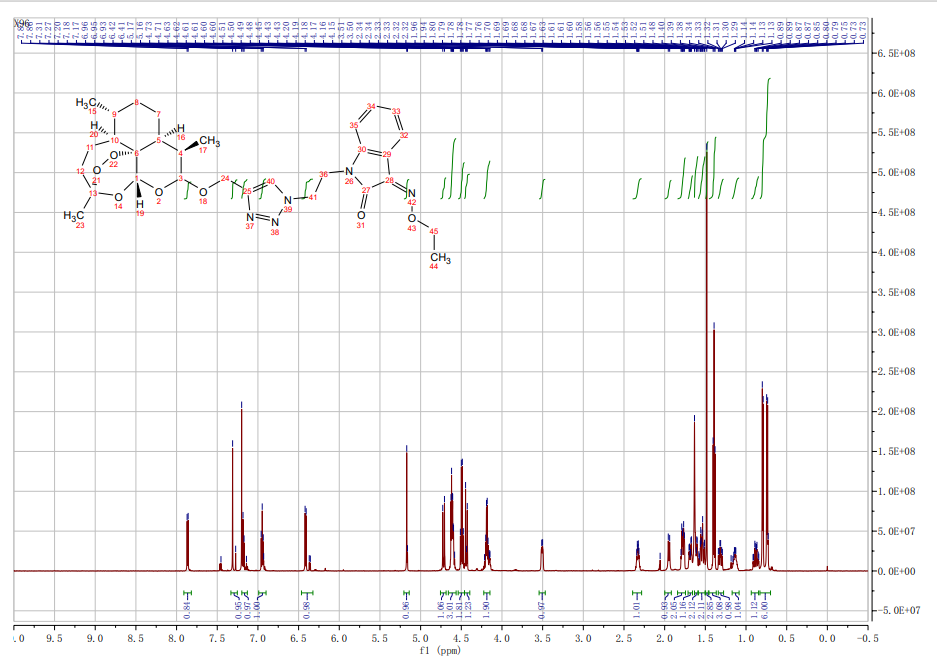


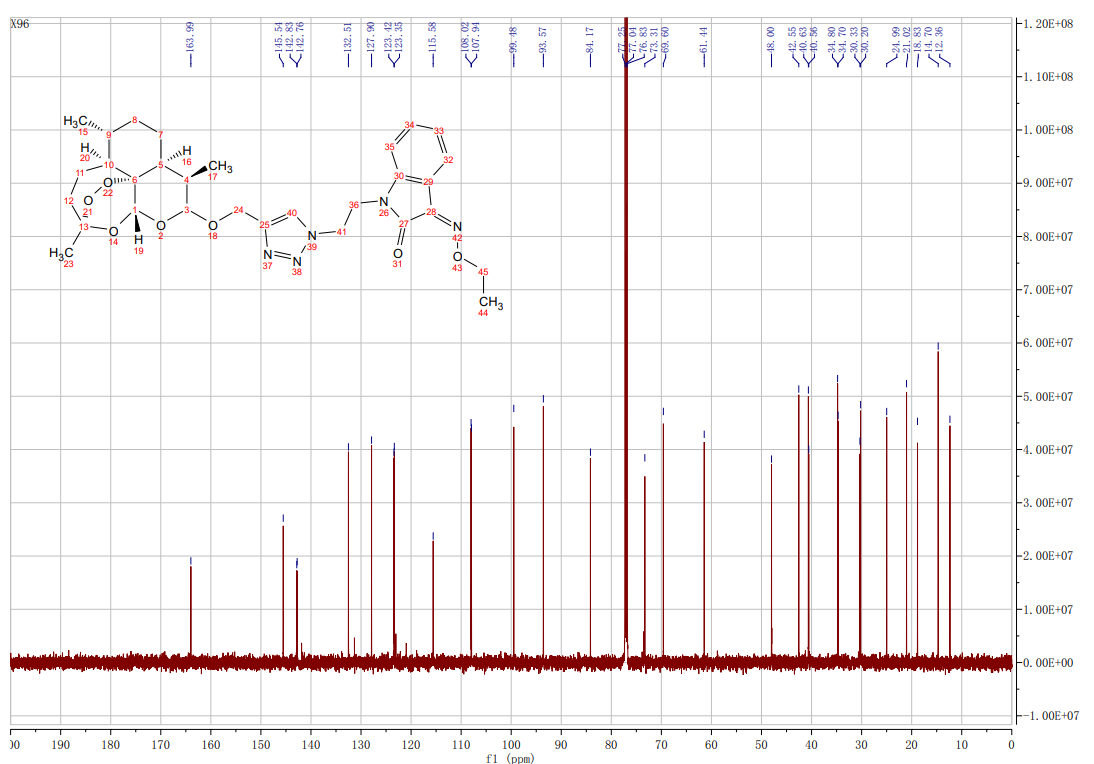


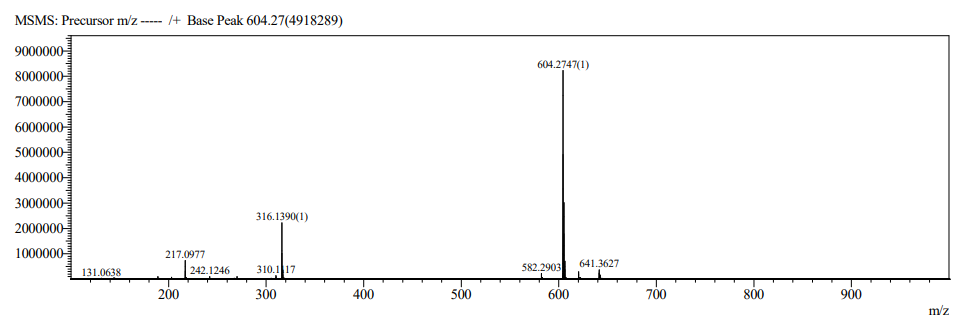


3-((benzyloxy)imino)-1-(2-(4-((((3*R*,5a*S*,6*R*,8a*S*,9*R*,12*R*,12a*R*)-3,6,9-trimethyldecahydro-12*H*-3,12-epoxy[1,2]dioxepino[4,3-i]isochromen-10-yl)oxy)methyl)-1*H*-1,2,3-triazol-1-yl)ethyl)indolin-2-one (**9e**)

Yellow solid, yield: 48%. ^1^H NMR (400 MHz, CDCl_3_) δ 0.72-0.88 (m, 7H), 1.12-1.14 (m, 1H), 1.29-1.32 (m, 1H), 1.48-1.56 (m, 5H), 1.59-1.62 (m, 2H), 1.66-1.70 (m, 2H), 1.75-1.79 (m, 2H), 2.33-2.34 (m, 1H), 3.50 (s, 1H), 4.18 (q, *J* = 4.0 Hz, 2H), 4.44 (d, *J* = 12.0 Hz, 1H), 4.63 (td, *J* = 4.0, 2.0 Hz, 2H), 4.72 (d, *J* = 8.0 Hz, 1H), 5.17 (s, 1H), 5.46 (s, 1H), 6.42 (d, *J* = 8.0 Hz, 1H), 6.90 (t, *J* = 8.0 Hz, 1H), 7.16 (t, *J* = 8.0 Hz, 1H), 7.30-7.39 (m, 6H), 7.82 (s, 1H). ^13^C NMR (100 MHz, CDCl_3_) δ 163.89, 145.55, 143.36, 142.88, 135.96, 132.74, 128.68, 128.62, 128.54, 128.15, 123.46, 124.43, 115.53, 108.02, 107.99, 99.50, 93.59, 84.17, 79.66, 69.61, 61.45, 47.98, 42.55, 40.62, 40.58, 34.80, 34.70, 30.33, 30.21, 24.99, 21.02, 18.83, 12.35. HRMS-ESI: m/z Calcd for C_35_H_41_N_5_O_7_Na [M+Na]^+^: 666.2899; Found: 666.2904.


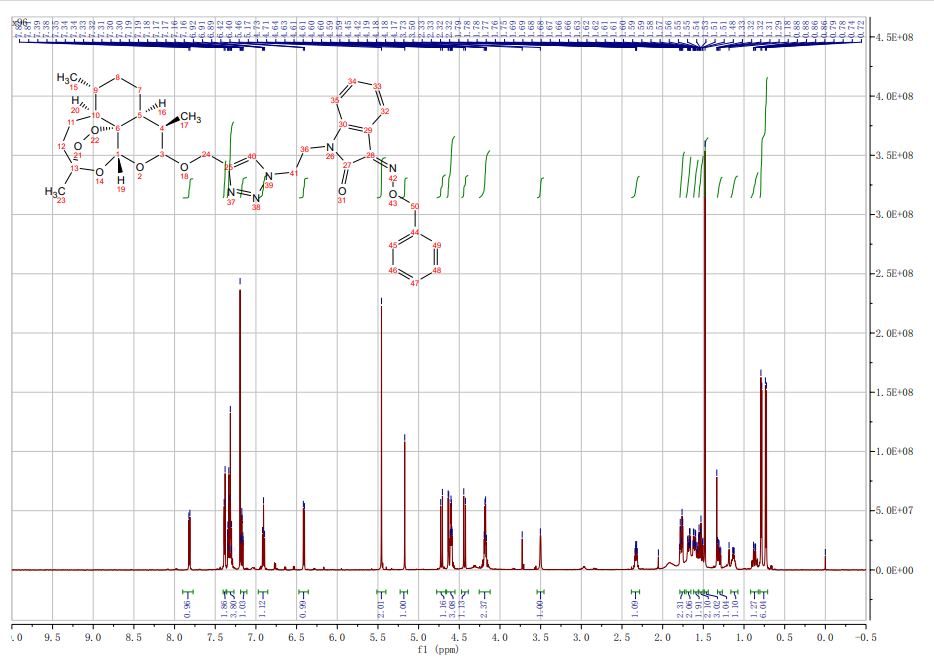


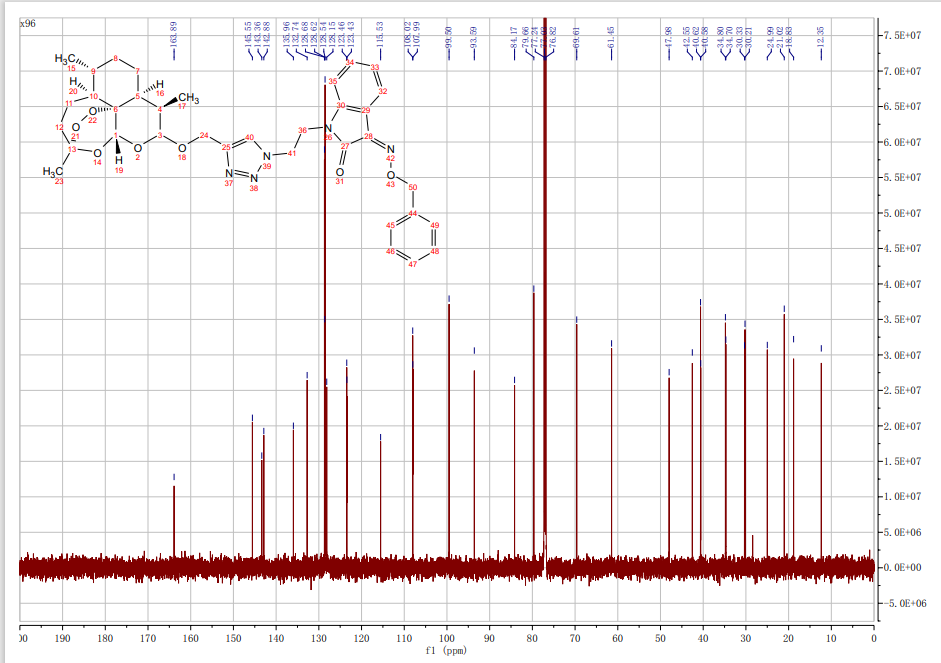


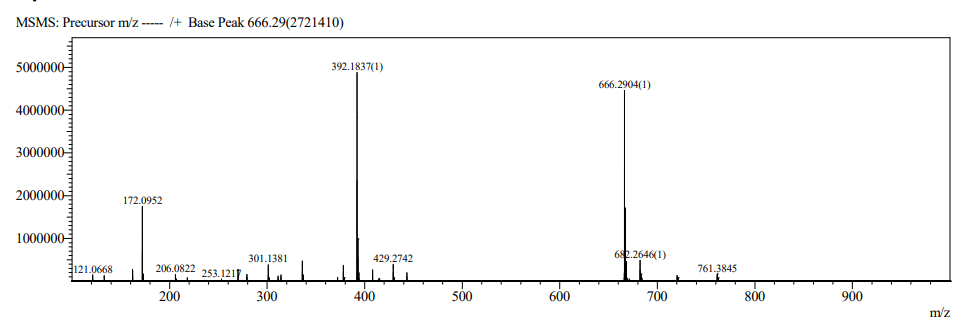


5-methoxy-3-(methoxyimino)-1-(2-(4-((((3*R*,5a*S*,6*R*,8a*S*,9*R*,12*R*,12a*R*)-3,6,9-trimethyldecahydro-12*H*-3,12-epoxy[1,2]dioxepino[4,3-i]isochromen-10-yl)oxy)methyl)-1*H*-1,2,3-triazol-1-yl)ethyl)indolin-2-one (**9f**)

Yellow solid, yield: 59%. ^1^H NMR (400 MHz, CDCl_3_) δ 0.74-0.91 (m, 7H), 1.11-1.16 (m, 1H), 1.29-1.34 (m, 1H), 1.48-1.55 (m, 5H), 1.60-1.63 (m, 1H), 1.67-1.70 (m, 1H), 1.76-1.80 (m, 2H), 1.98 (d, *J* = 4.0 Hz, 1H), 2.30-2.35 (m, 1H), 3.50 (d, *J* = 4.0 Hz, 1H), 3.69 (s, 3H), 4.12 (q, *J* = 4.0 Hz, 2H), 4.32 (s, 3H), 4.44 (d, *J* = 8.0 Hz, 1H), 4.58 (t, *J* = 4.0 Hz, 2H), 4.64 (d, *J* = 2.0 Hz, 1H), 4.72 (d, *J* = 8.0 Hz, 1H), 5.17 (s, 1H), 6.32 (d, *J* = 4.0 Hz, 1H), 6.72 (dd, *J* = 4.0, 2.0 Hz, 1H), 7.32 (s, 1H), 7.44 (d, *J* = 2.0 Hz, 1H). ^13^C NMR (100 MHz, CDCl_3_) δ 163.75, 156.02, 145.53, 143.28, 136.52, 123.45, 117.32, 116.05, 114.57, 108.54, 108.02, 99.48, 93.60, 84.15, 69.59, 65.01, 61.47, 55.88, 48.00, 42.53, 40.68, 40.62, 34.80, 34.68, 30.33, 30.22, 24.98, 20.99, 18.83, 12.30. HRMS-ESI: m/z Calcd for C_30_H_39_N_5_O_8_Na [M+Na]^+^: 620.2691; Found: 620.2674.


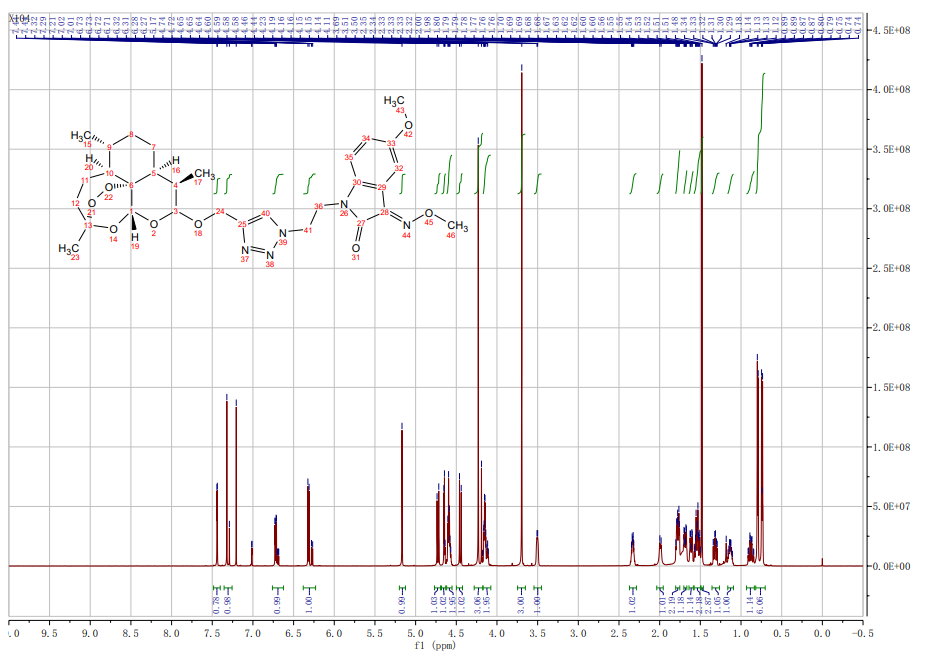


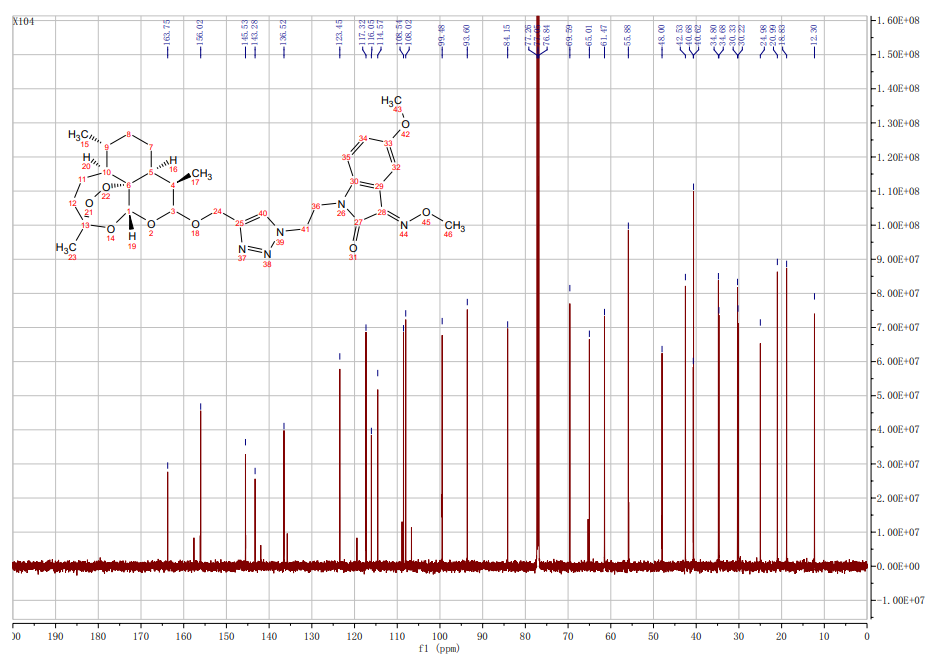


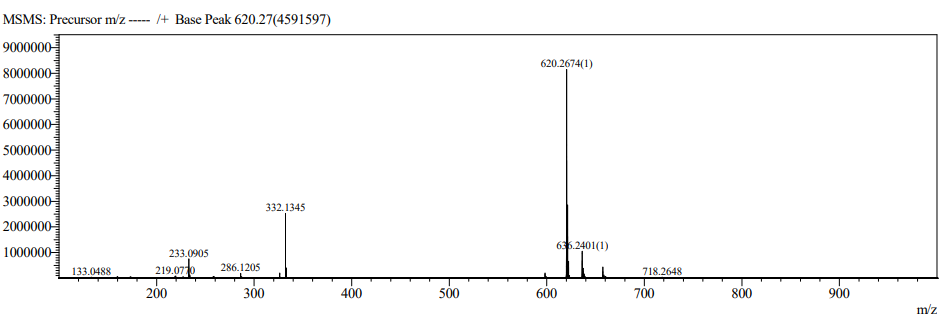


3-(ethoxyimino)-5-methoxy-1-(2-(4-((((3*R*,5a*S*,6*R*,8a*S*,9*R*,12*R*,12a*R*)-3,6,9-trimethyldecahydro-12*H*-3,12-epoxy[1,2]dioxepino[4,3-i]isochromen-10-yl)oxy)methyl)-1*H*-1,2,3-triazol-1-yl)ethyl)indolin-2-one (**9g**)

Yellow solid, yield: 42%. ^1^H NMR (400 MHz, CDCl_3_) δ 0.74-0.92 (m, 7H), 1.13-1.15 (m, 1H), 1.30-1.34 (m, 1H), 1.39 (t, *J* = 4.0 Hz, 3H), 1.48-1.54 (m, 5H), 1.56-1.61 (m, 2H), 1.66-1.69 (m, 1H), 1.76-1.79 (m, 2H), 1.90 (d, *J* = 4.0 Hz, 1H), 2.32-2.35 (m, 1H), 3.51 (d, *J* = 4.0 Hz, 1H), 3.70 (s, 3H), 4.16 (q, *J* = 4.0 Hz, 2H), 4.44 (d, *J* = 8.0 Hz, 1H), 4.50 (q, *J* = 4.0 Hz, 2H), 4.60 (td, *J* = 4.0, 2.0 Hz, 2H), 4.65 (d, *J* = 2.0 Hz, 1H), 4.72 (d, *J* = 8.0 Hz, 1H), 5.17 (s, 1H), 6.30 (d, *J* = 8.0 Hz, 1H), 6.72 (dd, *J* = 4.0, 2.0 Hz, 1H), 7.31 (s, 1H), 7.48 (d, *J* = 4.0 Hz, 1H). ^13^C NMR (100 MHz, CDCl_3_) δ 163.90, 155.99, 145.55, 143.11, 136.42, 123.44, 116.87, 116.21, 114.73, 108.42, 108.02, 99.51, 93.60, 84.17, 73.35, 69.62, 61.49, 55.85, 48.06, 42.55, 40.69, 40.64, 34.80, 34.70, 30.33, 30.23, 24.99, 21.00, 18.83, 14.71, 12.32. HRMS-ESI: m/z Calcd for C_31_H_41_N_5_O_8_Na [M+Na]^+^: 634.2848; Found: 634.2839.


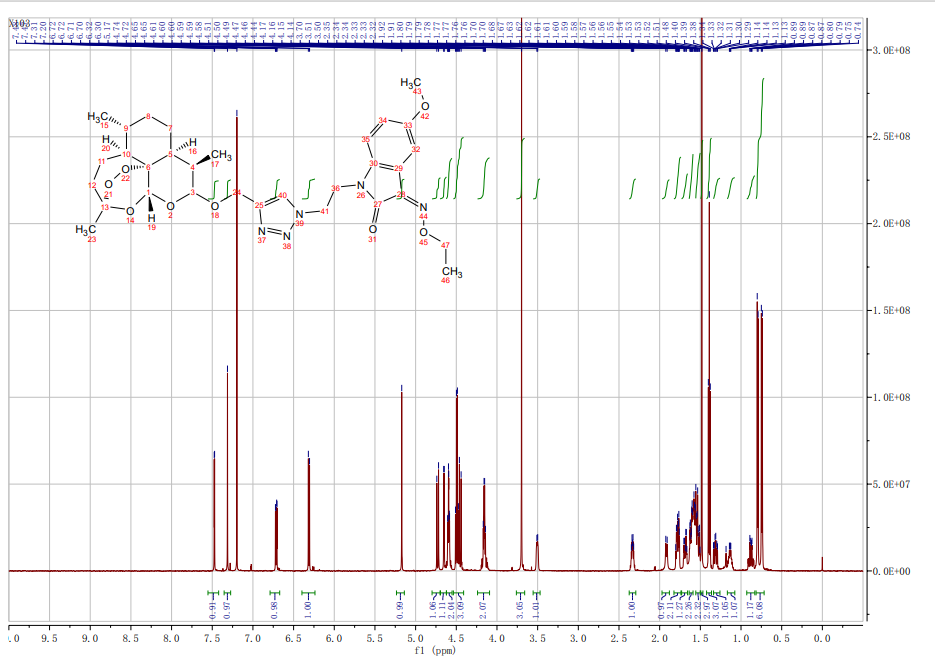


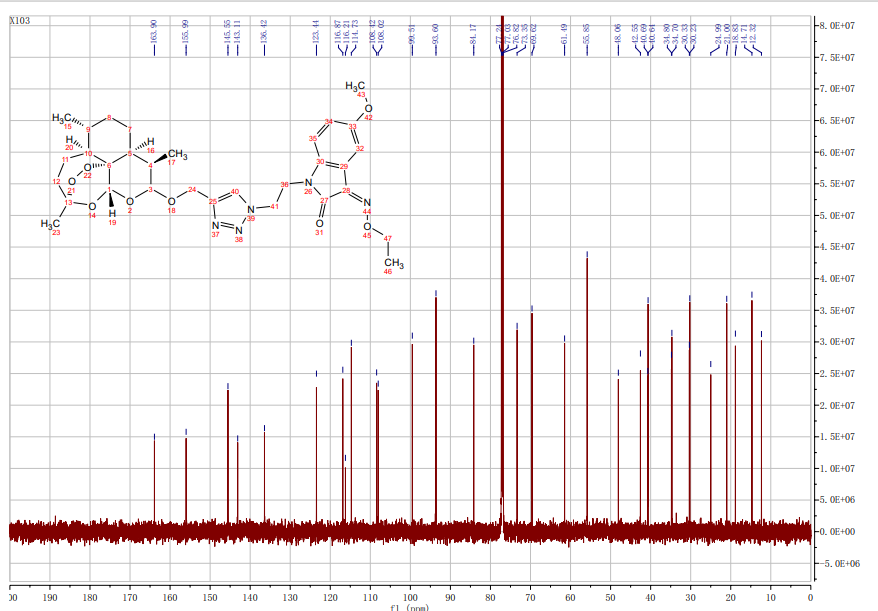


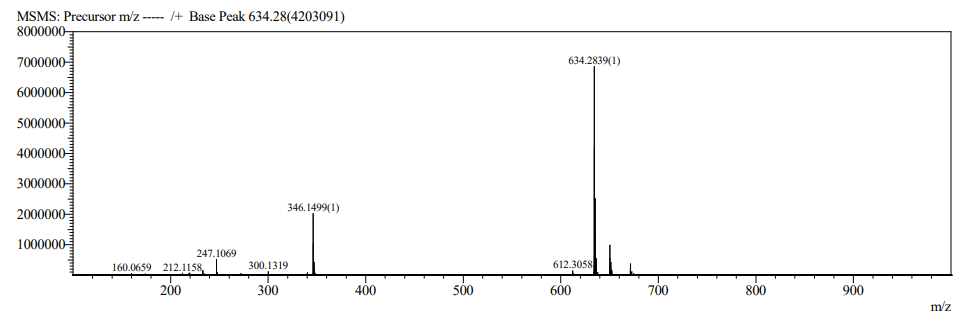


3-((benzyloxy)imino)-5-methoxy-1-(2-(4-((((3*R*,5a*S*,6*R*,8a*S*,9*R*,12*R*,12a*R*)-3,6,9-trimethyldecahydro-12*H*-3,12-epoxy[1,2]dioxepino[4,3-i]isochromen-10-yl)oxy)methyl)-1*H*-1,2,3-triazol-1-yl)ethyl)indolin-2-one (**9h**)

Yellow solid, yield: 41%. ^1^H NMR (400 MHz, CDCl_3_) δ 0.73-0.90 (m, 7H), 1.12-1.15 (m, 1H), 1.28-1.33 (m, 1H), 1.48-1.56 (m, 5H), 1.60-1.62 (m, 2H), 1.66-1.69 (m, 1H), 1.76-1.79 (m, 2H), 1.90 (d, *J* = 8.0 Hz, 1H), 2.31-2.34 (m, 1H), 3.50 (d, *J* = 4.0 Hz, 1H), 3.62 (s, 1H), 4.16 (q, *J* = 4.0 Hz, 2H), 4.44 (d, *J* = 8.0 Hz, 1H), 4.58 (td, *J* = 4.0, 2.0 Hz, 2H), 4.66 (d, *J* = 4.0 Hz, 1H), 4.72 (d, *J* = 8.0 Hz, 1H), 5.17 (s, 1H), 5.46 (s, 1H), 6.30 (d, *J* = 4.0 Hz, 1H), 6.70 (dd, *J* = 4.0, 2.0 Hz, 1H), 7.28-7.39 (m, 6H), 7.44 (d, *J* = 2.0 Hz, 1H). ^13^C NMR (100 MHz, CDCl_3_) δ 163.79, 156.01, 145.56, 143.70, 136.54, 135.97, 128.67, 128.61, 128.44, 123.45, 117.25, 116.13, 114.78, 108.52, 108.02, 99.51, 93.62, 84.16, 79.61, 69.62, 61.49, 55.77, 48.04, 42.54, 40.71, 40.63, 34.80, 34.69, 20.33, 30.23, 24.98, 21.00, 18.83, 12.32. HRMS-ESI: m/z Calcd for C_36_H_43_N_5_O_8_Na [M+Na]^+^: 696.3004; Found: 696.2981.


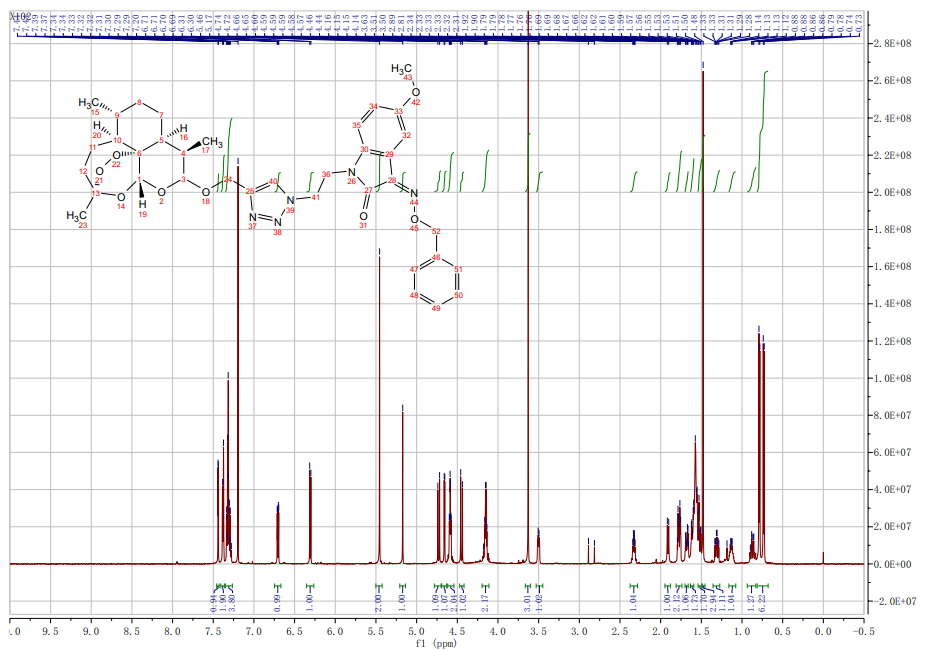


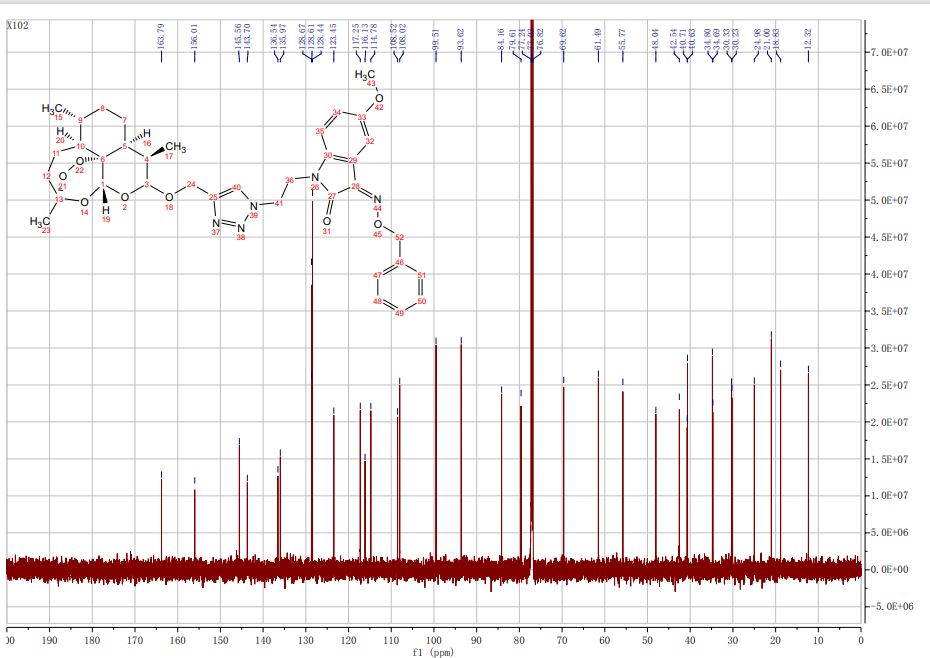


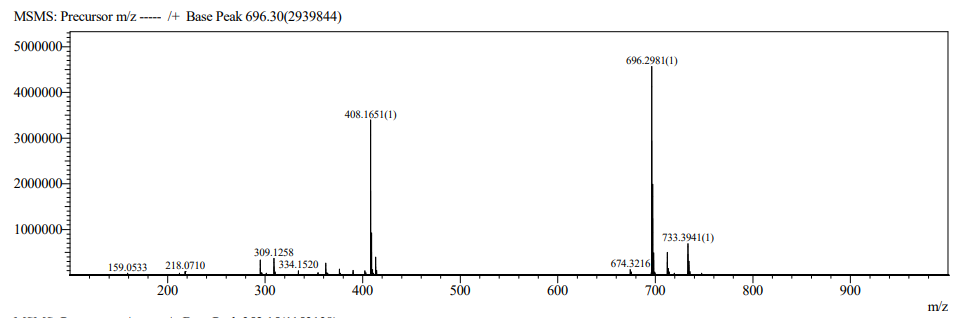


5-fluoro-3-(hydroxyimino)-1-(2-(4-((((3*R*,5a*S*,6*R*,8a*S*,9*R*,12*R*,12a*R*)-3,6,9-trimethyldecahydro-12*H*-3,12-epoxy[1,2]dioxepino[4,3-i]isochromen-10-yl)oxy)methyl)-1*H*-1,2,3-triazol-1-yl)ethyl)indolin-2-one (**9i**)

Yellow solid, yield: 67%. ^1^H NMR (400 MHz, CD_3_OD) δ 0.72-0.88 (m, 7H), 1.11-1.13 (m, 1H), 1.19-1.21 (m, 1H), 1.37-1.41 (m, 4H), 1.48-1.59 (m, 3H), 1.64-1.69 (m, 2H), 1.75-1.78 (m, 1H), 2.28-2.30 (m, 1H), 3.39 (d, *J* = 2.0 Hz, 1H), 4.16 (t, *J* = 4.0 Hz, 1H), 4.40 (d, *J* = 8.0 Hz, 1H), 4.60-4.67 (m, 4H), 5.16 (s, 1H), 6.64 (dd, *J* = 4.0, 2.0 Hz, 1H), 6.98 (td, *J* = 8.0, 2.0 Hz, 1H), 7.66 (dd, *J* = 8.0, 2.0 Hz, 1H), 7.80 (s, 1H). ^13^C NMR (100 MHz, CD_3_OD) δ 164.50, 159.72 (*J* = 198.75 Hz), 144.84, 142.97, 138.62, 124.46, 117.69, 117.53, 116.17, 114.39, 114.21, 109.14, 109.09, 107.92, 98.97, 93.51, 83.73, 68.64, 60.32, 42.10, 40.67, 40.03, 34.52, 30.24, 30.13, 24.80, 20.02, 17.88, 11.28. HRMS-ESI: m/z Calcd for C_28_H_34_FN_5_O_7_Na [M+Na]^+^: 594.2335; Found: 594.2330.


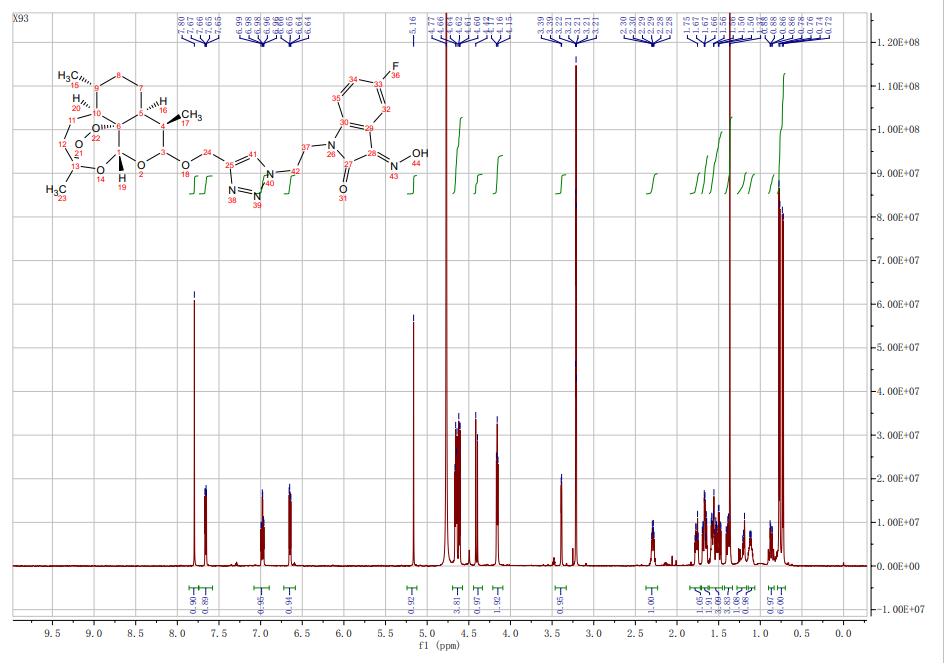


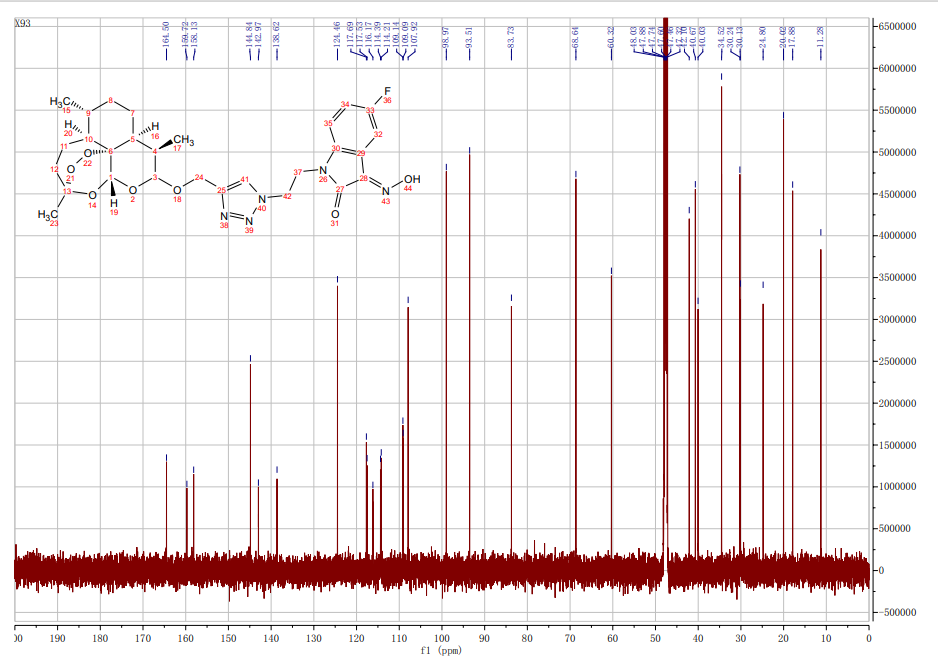


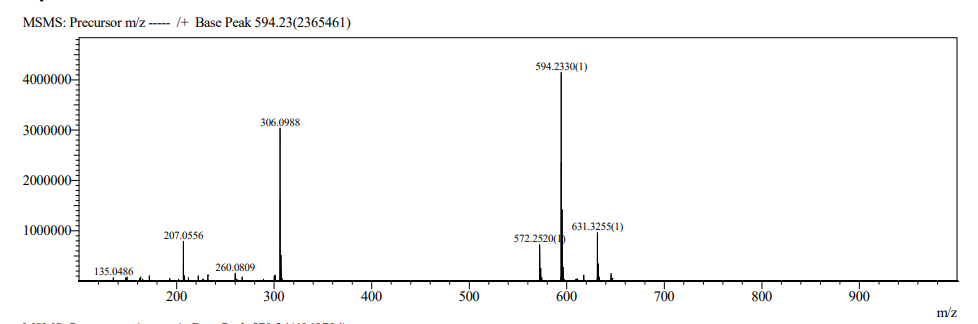


5-fluoro-3-(methoxyimino)-1-(2-(4-((((3*R*,5a*S*,6*R*,8a*S*,9*R*,12*R*,12a*R*)-3,6,9-trimethyldecahydro-12*H*-3,12-epoxy[1,2]dioxepino[4,3-i]isochromen-10-yl)oxy)methyl)-1*H*-1,2,3-triazol-1-yl)ethyl)indolin-2-one (**9j**)

Yellow solid, yield: 52%. ^1^H NMR (400 MHz, CDCl_3_) δ 0.74-0.90 (m, 7H), 1.13-1.15 (m, 1H), 1.30-1.33 (m, 1H), 1.48-1.56 (m, 6H), 1.60-1.63 (m, 1H), 1.68-1.71 (m, 1H), 1.76-1.80 (m, 2H), 1.88 (d, *J* = 8.0 Hz, 1H), 2.33-2.35 (m, 1H), 3.50 (dd, *J* = 8.0, 4.0 Hz, 1H), 4.15-4.25 (m, 4H), 4.44 (d, *J* = 8.0 Hz, 1H), 4.60 (t, *J* = 4.0 Hz, 1H), 4.65 (d, *J* = 4.0 Hz, 1H), 4.74 (d, *J* = 8.0 Hz, 1H), 5.18 (s, 1H), 6.34 (dd, *J* = 4.0, 2.0 Hz, 1H), 6.90 (t, *J* = 4.0 Hz, 1H), 7.30 (s, 1H), 7.58 (dd, *J* = 4.0, 2.0 Hz, 1H). ^13^C NMR (100 MHz, CDCl_3_) δ 174.32, 159.74 (*J* = 198.75 Hz), 145.63, 123.46, 118.98, 118.54, 108.03, 99.48, 93.95, 84.15, 69.63, 65.25, 61.48, 48.00, 42.52, 40.78, 40.62, 34.82, 30.32, 30.22, 24.98, 20.98, 18.83, 12.27. HRMS-ESI: m/z Calcd for C_29_H_36_FN_5_O_7_Na [M+Na]^+^: 608.2491; Found: 608.2483.


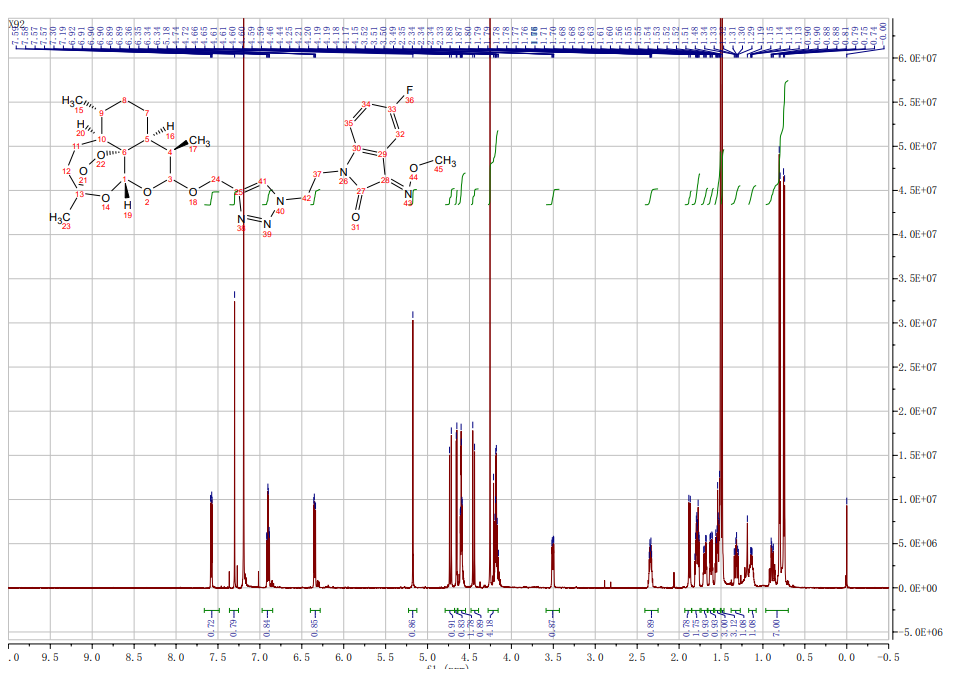


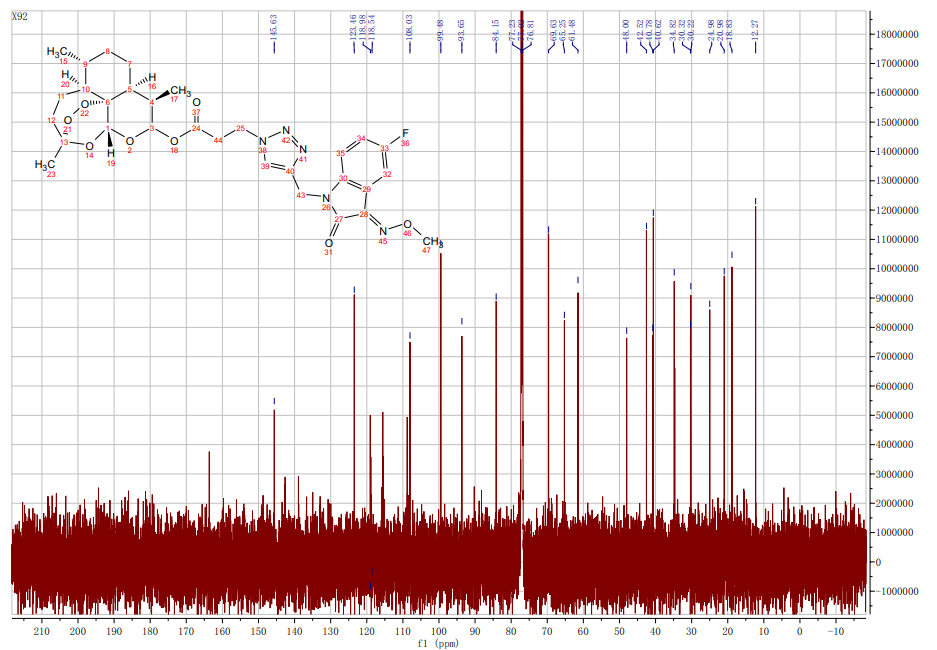


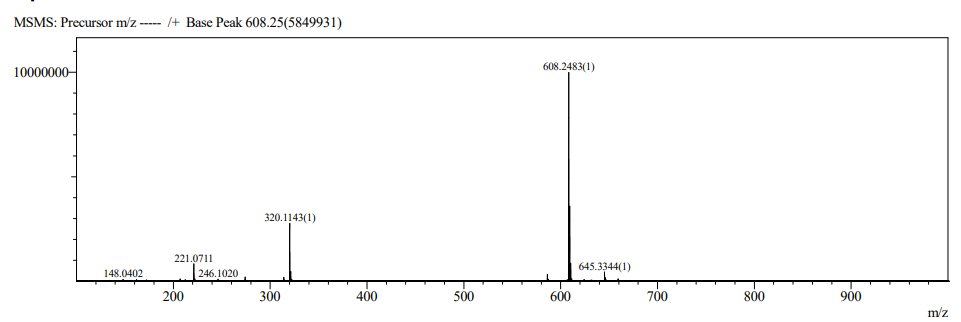


3-(ethoxyimino)-5-fluoro-1-(2-(4-((((3*R*,5a*S*,6*R*,8a*S*,9*R*,12*R*,12a*R*)-3,6,9-trimethyldecahydro-12*H*-3,12-epoxy[1,2]dioxepino[4,3-i]isochromen-10-yl)oxy)methyl)-1*H*-1,2,3-triazol-1-yl)ethyl)indolin-2-one (**9k**)

Yellow solid, yield: 33%. ^1^H NMR (400 MHz, CDCl_3_) δ 0.73-0.90 (m, 7H), 1.16-1.18 (m, 1H), 1.32-1.33 (m, 1H), 1.40 (t, *J* = 4.0 Hz, 3H), 1.48-1.56 (m, 5H), 1.60-1.70 (m, 3H), 1.76-1.80 (m, 2H), 1.92 (d, *J* = 8.0 Hz, 1H), 2.33-2.35 (m, 1H), 3.49-3.51 (m, 1H), 4.17-4.20 (m, 2H), 4.44 (d, *J* = 8.0 Hz, 1H), 4.50 (q, *J* = 4.0 Hz, 2H), 4.60 (t, *J* = 4.0 Hz, 1H), 4.66 (d , *J* = 4.0 Hz, 1H), 4.74 (d, *J* = 8.0 Hz, 1H), 5.18 (s, 1H), 6.34 (dd, *J* = 4.0, 2.0 Hz, 1H), 6.88 (t, *J* = 4.0 Hz, 1H), 7.31 (s, 1H), 7.60 (dd, *J* = 4.0, 2.0 Hz, 1H). ^13^C NMR (100 MHz, CDCl_3_) δ 163.77, 159.68 (*J* = 200.00 Hz), 145.60, 142.44, 138.82, 123.49, 118.83, 118.67, 116.03, 115.43, 115.26, 108.72, 108.66, 108.03, 99.46, 93.64, 84.14, 73.69, 69.61, 61.46, 48.05, 42.51, 40.77, 40.61, 34.82, 34.68, 30.33, 30.22, 24.98, 20.98, 18.82, 14.69, 12.28. HRMS-ESI: m/z Calcd for C_30_H_38_FN_5_O_7_Na [M+Na]^+^: 622.2648; Found: 622.2630.


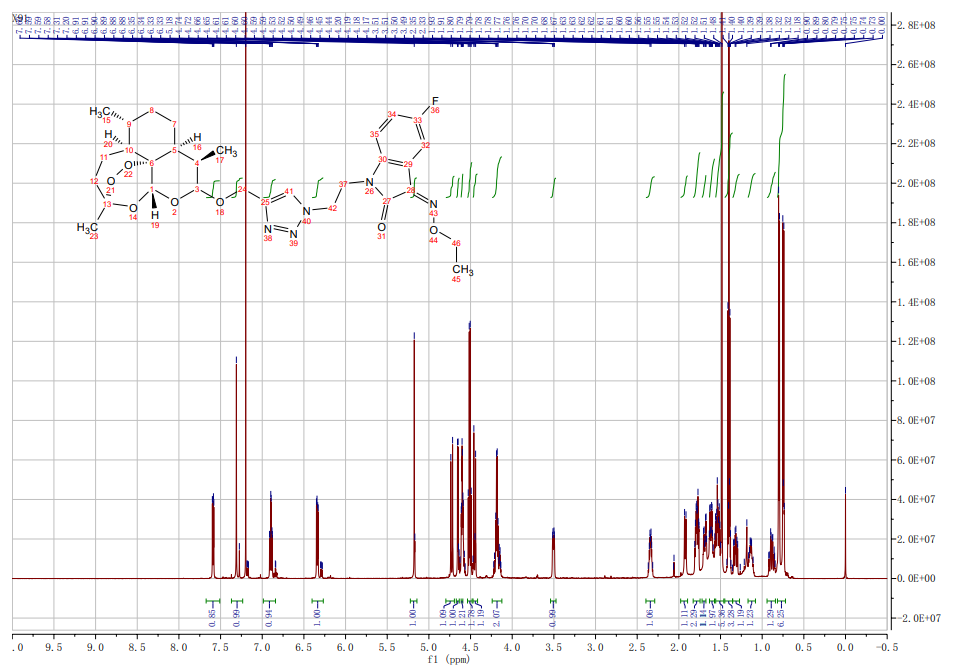


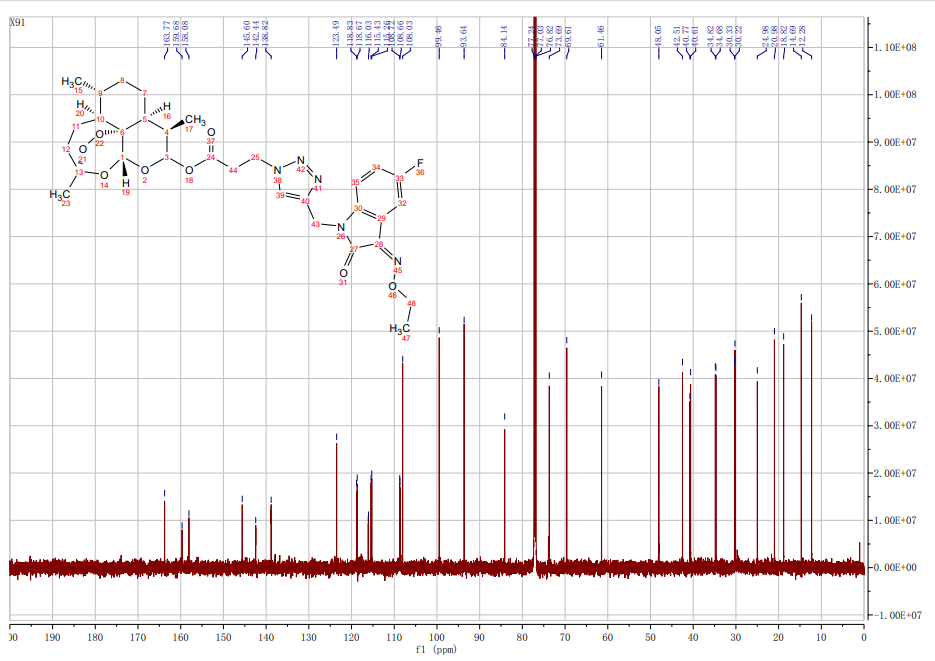


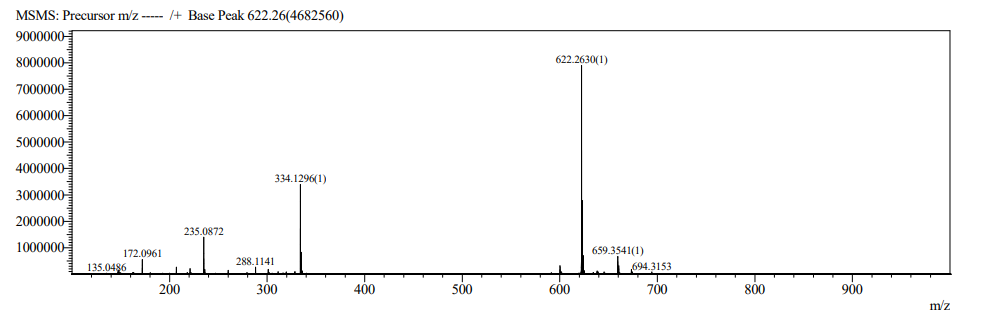

Supplement: Supplementary file 1 [file DataSheet1.docx]
